# Integrating heterogeneous data to address endemic diseases in broiler production: insights from a Polish case study

**DOI:** 10.1186/s12917-026-05341-x

**Published:** 2026-03-09

**Authors:** Camille Delavenne, Marcin Śmiałek, Jacobus Joannes (Sjaak) de Wit, Céline Faverjon

**Affiliations:** 1 EpiMundi, Lyon, France; 2 Faculty of Veterinary Medicine, Utrecht University, Utrecht, The Netherlands; 3 Faculty of Veterinary Medicine, University of Warmia and Mazury, Olsztyn, Poland; 4 SLW BIOLAB, Ostróda, Poland; 5 Royal GD, Deventer, The Netherlands

**Keywords:** Endemic disease, Poultry, Data reuse, Cluster analysis, Health, Production performance

## Abstract

**Background:**

Endemic contagious diseases in broilers have a significant impact on production performance. However, endemic contagious diseases are multifaceted and complex. They are rarely monitored on a large scale. This complexity hinders their mitigation, as timely information about their distribution and knowledge about their impact on production performance is scarce.

This study aimed to evaluate whether data routinely produced by the Polish broiler industry, the first European meat producer, could be reused to generate knowledge about those diseases and provide stakeholders with contextual information to improve their disease management.

**Results:**

The study reused a dataset collected by a large producer and a veterinary laboratory, which implemented a screening program at the end of the production cycle. The high-dimensional dataset covered 115 ‘screened flocks’ produced between 2018 and 2023 across Poland. The information was dispersed across 75 variables on production indicators, health indicators, necropsy lesions, and a list of evidence of infection or infestation by a diverse range of aetiological agents (bacterial, viral, and Eimeria) and regrouped into 8 themes. The ‘screened flocks’, despite strong production performance indicators, exhibited a high mortality rate (mean of 6.01%) and a wide range of pathogens (19 bacteria, 3 viruses, and Eimeria infestation observed). The cluster analysis, after identifying two outliers, defined three flock profiles, linking the observation variables (health, production indicator, necropsy lesions) to the aetiological agents. ‘Screened flocks’ from the first cluster (27 flocks) were described as a flock with high rates of fibrinous lesions, with a high condemnation rate associated with the identification of E. coli. The second cluster (64 flocks) was defined by high production performances but also higher rates of femoral head necrosis. The ‘screened flocks’ from the last cluster (22 flocks) exhibited lower production performance, indicating strong Eimeria spp. infestation and circulation of avian metapneumovirus.

**Conclusion:**

The study is an example of how high-dimensional data produced by the broiler industry can be reused and integrated to create contextual knowledge for farmers and veterinarians about endemic contagious diseases. Access to this timely contextual knowledge is essential to enhance disease prevention and management efforts for farmers, veterinarians, and other stakeholders in the broiler industry.

**Supplementary Information:**

The online version contains supplementary material available at 10.1186/s12917-026-05341-x.

## Background

Since the 1970s, the global poultry industry has expanded continuously to meet a growing demand for poultry meat [1]. This production growth could not have been achieved without a steep increase in production efficiency, driven by growing technical capacities in genetics, housing, nutrition, biosecurity, and health management [2]. Despite these achievements, the broiler production sector still faces multiple challenges, including increased resource competition, growing threats from emerging infectious diseases, and higher consumer expectations regarding food safety, animal welfare, and environmental impacts [2–4]. Addressing these challenges requires the industry to evolve while maintaining low production costs to meet market demand. One of the main areas for improving production efficiency is mitigating the impact of endemic contagious diseases [5]. However, these diseases are multifaceted and complex, involving interactions among pathogens, animal genetics, and the environment, and their impacts vary across diverse contexts [2, 6]. This complexity hinders access to up-to-date information on the status and production impact of poultry-endemic contagious diseases [6], despite their necessity to address the sector’s persistent challenges.

Implementing epidemiological studies on endemic contagious diseases and their impacts can be expensive, time-consuming, and often too slow to keep pace with context-specific trends [7]. Using pre-existing data sources to generate more timely and targeted information for the industry has been described as a cost-effective solution compared to some traditional approaches [3, 7]. In the broiler production sector, data collection and digitalisation have also escalated with the expansion of technical capacities. Indeed, stakeholders across the value chain increasingly use data to support their activities with different intensity levels [8]. These data offer timely insight into farm and poultry production, as well as the activities of other value chain stakeholders, which could be key to optimising endemic contagious disease management [9, 10]. Furthermore, routinely reusing and integrating production and health data could provide data-driven information to prioritise investments and action [6, 11].

Such data health solutions, based on complex multivariate analyses, are being developed in precision livestock farming to capitalise on this opportunity [8, 10]. However, they primarily focus on collecting new data rather than integrating existing data sources [7, 9]. These approaches thus do not address the specific issue encountered by poultry producers, where data about a flock can be dispersed across multiple actors throughout the production chain (e.g., flock manager, slaughterhouse, feed mill, veterinarian, diagnostic laboratory, and competent governmental authorities), even in the context of highly integrated production systems. Furthermore, in broilers, investigating contagious diseases solely using reused stakeholder data remains rare, with many studies focusing on production, slaughter or antimicrobial use data [12–15]. To build tools or conduct research that reuse these integrated data, industry and researchers face various challenges associated with data ownership, accessibility, quality, and integration [3]. Furthermore, farmers’ investment in and adoption of digital technologies have been hindered by high costs, a lack of comprehensive technological offerings, and concerns that these technologies will not harm them, necessitating strong collaboration to bridge the gap [10, 16]. Before farmers invest in technologies that enable data integration for health purposes, researchers need to demonstrate to producers the value of data reuse and integration in terms of support for production and health management [3, 17, 18].

In this context, Poland is Europe’s largest poultry meat producer, with 2,746 thousand tonnes of meat produced in 2023 [19, 20]. Polish producers suffer the same constraints as the rest of the industry but operate in an environment characterised by rising production costs and overall volatility of raw material prices [21]. Moreover, the Polish broiler production is not highly integrated despite its size [21]. This fragmented structure fosters a highly competitive environment, breeding distrust and creating heterogeneous data flows and hindering the integration of data across the value chain. Indeed, comprehensive information about poultry endemic contagious diseases in Poland, such as coccidiosis, infectious bronchitis virus (IBV), infectious bursal disease virus (IBDV), or avian metapneumovirus (aMPV), is scarce and fragmented. Polish broiler production is therefore a good case study to demonstrate the current value of the fragmented data produced by farmers and to better understand contagious diseases affecting those flocks.

The present study aims to improve our understanding of the major endemic contagious diseases affecting the Polish production system by reusing routine high-dimensional data from broiler producers and a diagnostic laboratory. To achieve this, the study describes the dataset using common exploratory data analysis techniques and identifies the predominant health and production patterns amongst the studied broiler flocks using clustering techniques. It discusses their potential association with specific aetiological agents, considering plausible biological mechanisms to define strategies to enhance animal production, health, and welfare. Furthermore, it assesses the quality of the information produced. Finally, it discusses its application to Polish broiler stakeholders in a context where data integration and reuse for health management purposes are relatively uncommon.

## Methodology

### Data sources

**Fig. 1:**
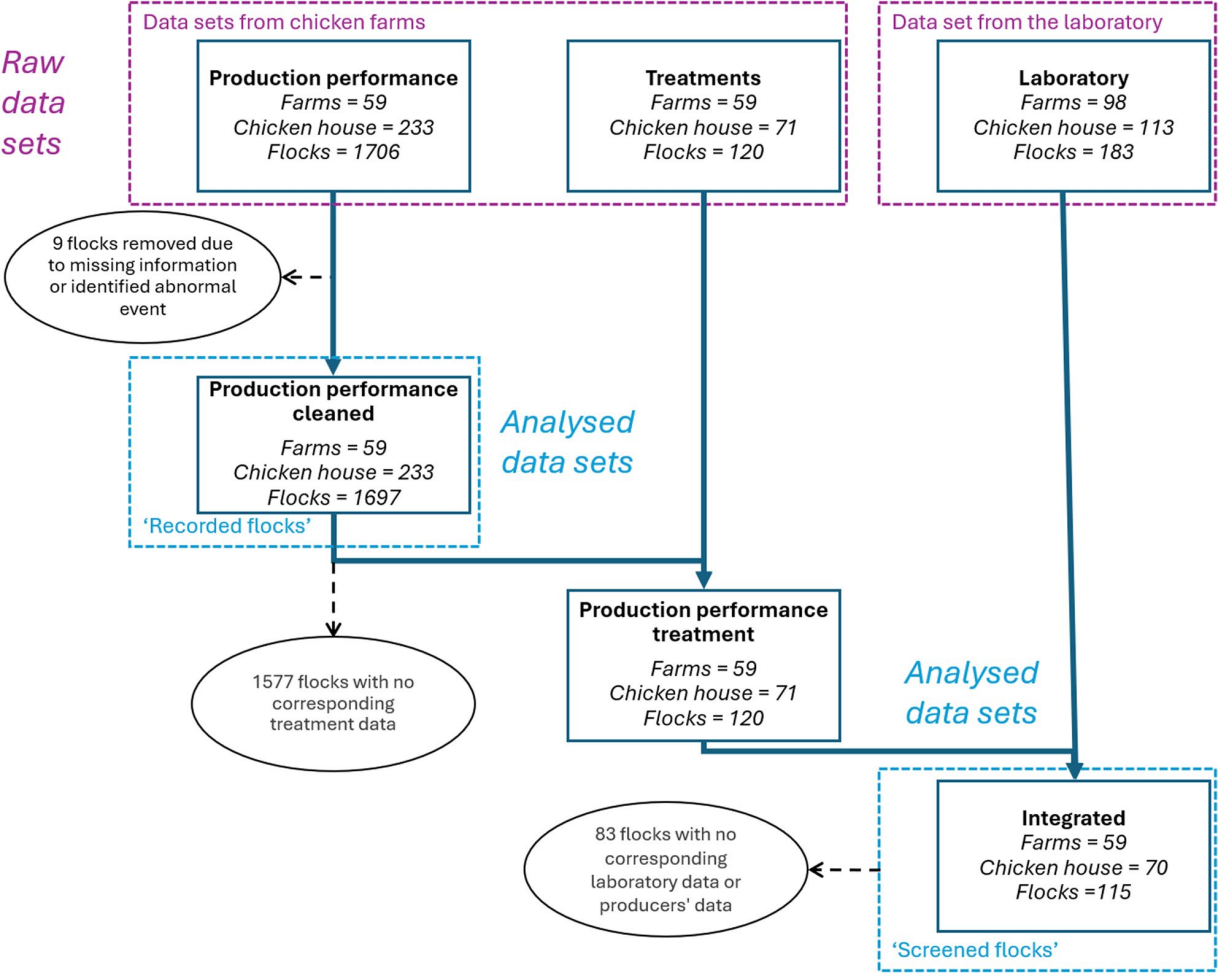
Data cleaning and integration process

All the data came from pseudo-anonymised poultry farms growing Ross308 broilers in the northeast region of Poland between 2018 and 2023. They were made of two distinct datasets: the “chicken farm” dataset provided by poultry producers, and the “laboratory” datasets provided by a veterinary diagnostic laboratory (see Fig. 1).

The “chicken farm” dataset included standard indicators used to evaluate performance of broiler flocks (i.e., mortality, death on arrival at the slaughterhouse, mean age at slaughter, foot-pad lesion scores, etc.) and information about vaccinations and antibiotic treatments administered during the flock production cycle (i.e., name of the product or active substance, day of treatment, and route of administration). This dataset collected information from 59 farms, but the amount of information available for each farm varied between one and 31 production cycles. Moreover, the treatment data was available for less than 7% of the flocks with production performance data.

The “laboratory” dataset was related to tests performed during the last week of production cycles as part of a screening program proposed to farmers by the veterinary diagnostic laboratory. This dataset collected information from 183 flocks coming from 98 farms. Necropsy observations and bacteriological, parasitological, and virological test results were available for five chickens randomly sampled per flock. On the same day, blood/serum from 23 birds of each screened flock was also randomly collected to be analysed serologically for three diseases (the same as the one investigated in the virological test). Technical details on the laboratory test used are available in Additional file 1.

### Data processing

Both datasets were made of multiple Excel spreadsheets. The data was processed and integrated following the process described in Fig. 1. The datasets were combined using the chicken house ID, flock production date (start and end), and laboratory analysis date. A laboratory result was attached to the flock if it occurred during the production interval or seven days after. Among the laboratory results, the unmatched results (either from farms without production data or from production cycles before or after the shared historical data) were not used in the analysis. The ‘recorded flocks’ are all the flocks used in the study. The 115 ‘screened flocks’ are a sample of the ‘recorded flocks’, defined as the flocks with both laboratory results and production performance data available. These terms are used in Fig. 1 and the rest of the paper.

The data was then processed into 75 variables grouped into eight themes. Half of the themes were related to the data provided by the broiler producers (i.e., ‘Context’, ‘Economic indicator’, ‘Production performance’, ‘Health and Welfare’), three to the data provided by the laboratory (i.e., ‘Necropsy lesions’, ‘Bacteria status’, ‘Eimeria status’) and one (i.e. ‘Viral status’) was based on a combination of information extracted from the two datasets. The themes and their associated variables are described below and summarised in Table 1.

**Table 1:** Description of the integrated data after cleaning and processing into eight themes and 75 variables

Themes	Variable	Type*	Definition
Contextual	Season	Spring	Production mainly occurred from the 1st of March until the 31st of May
		Summer	Production mainly occurred from the 1st of June until the 31st of August
		Autumn	Production mainly occurred from the 1st of September until the 30th of November
		Winter	Production mainly occurred from the 1st of December until the 28th of February
	Region	PL9	Farm’s postal code was included in region PL9 -Masovian voivodeship
		PL6	Farm’s postal code was included in region PL6- Północny region
		PL8a	Farm’s postal code was included in southern region PL8 - Lublin Voivodeship
		PL8b	Farm’s postal code was included in northern region PL8 - Podlaskie Voivodeship
	Farm size	Small	Farm with 1 or 2 chicken houses
		Large	Farm with over two chicken houses
	ATB use	Y	Antibiotics were used during the production cycle
		N	Antibiotics were used during the production cycle
Economic	EPEF	*Continuous*	European production efficiency factor
Production performance	Mean age at slaughter	*Continuous*	Mean age at slaughter of birds in days
	Mean weight at slaughter	*Continuous*	Mean measured weight of birds slaughtered in Kg
	FCR (Feed conversion rate)	*Continuous*	Total weight of feed distributed to the flock divided by the total weight of birds sold
Health and welfare	Mortality	*Interval*	Number of chicks ordered at the hatchery minus the number of birds sold to the slaughterhouse divided by the number of birds sold (%)
	DOA (Dead on arrival)	*Interval*	Number of birds dead on arrival (transport) divided by the number of birds sold (%)
	Condemnation	*Interval*	Number of birds condemned divided by the number of birds sold (%)
	FPLS (Foot-pad lesion score)	*Interval*	Sum of the weighted product of birds with foot-pad lesions of grade 1 (*0.5) and grade 2 (* 2) amongst a hundred randomly sampled birds
Necropsy lesion	37 variables listed in Table 4	N	The validated lesion wasn’t observed in the ‘screened flocks’
		Y	The validated lesion was observed in at least one bird in the ‘screened flock’
Bacteria status	19 variables listed in Table 5	N	The bacteria group wasn’t identified in the ‘screened flocks’
		Y	The bacteria group was identified in at least one bird in the ‘screened flocks’
Eimeria status	Variable defined for 4 intestinal sections	No	No evidence of circulation - the sum of all grades of screened birds equals to 0
		Low	Low evidence of circulation - the sum of all grades of screened birds is between 1 and 3
		High	High evidence of circulation- the sum of all grades of screened birds is over 3
Virus circulation	Variable defined for 3 endemic viral diseases.	No	No virus field strain circulation; see Additional file 2
		SUS	Suspicion of any virus field strain circulation, see Additional file 2
		HSUS	High suspicion of IBV virus field strain circulation; see Additional file 2
		Yes	Confirmed field strain circulation; see Additional file 2

The full integrated data was only available for the ‘screened flocks’. The column in the data table named ‘Type’ provides the categories of the nominal variables or the type (continuous, interval) of the quantitative variables when underlined

#### Theme: context

Four contextual variables were defined: ‘season’ and ‘region’ of production, ‘farm size’ and Antibiotic use (‘ATB use’). The season of production was determined based on the dates of flock production. A four-season model was used, with spring beginning on March 1st, summer on June 1st, autumn on September 1st, and winter on December 1st. Flocks with a production period overlapping two different seasons were assigned to the season that overlapped the most with the production period (most days). The production region was based on postal codes and the definition of the major socio-economic regions as defined in the European nomenclature of territorial units for statistics (NUTS 1), except for one, the NUTS coded ‘PL8’, which was divided into two parts: north and south. The farm size was defined based on the number of chicken houses [1–2, or over 2]. Finally, flocks were categorised based on the use or non-use of antibiotics during the production cycle.

#### Theme: production performance

Three flock performance variables were identified and based on existing indicators which had been defined and used by the poultry farmer to monitor their flocks: ‘mean age at slaughter’ in days, ‘mean weight at slaughter’ in Kg and the ‘Feed Conversion Rate’ (‘FCR’). The ‘mean age at slaughter’ and ‘mean weight at slaughter’ were defined in the dataset and, in the usual broiler value chain, are calculated by the slaughterhouse as the geometric mean of all broilers’ age and weight sent to the slaughterhouse during the flock’s thinning and final clearance. The ‘FCR’ is a standard indicator calculated based on available information in the dataset and is defined as the total weight of feed (Kg) provided to the flocks divided by the total weight of birds sold at the slaughterhouse.

#### Theme: health and welfare

Four health and welfare variables were defined. ‘Mortality’ was expressed as a percentage and defined as the difference between the number of chicks bought at the hatchery and the number of birds sold at slaughter (before death in transport and condemnation) divided by the number of birds sold at slaughter. The ‘DOA’ (Dead on arrival) percentage was defined as the number of birds dead on arrival at slaughter (during transport) divided by the total of birds sold at slaughter. The percentage of ‘condemnation’ was defined as the number of birds condemned at the slaughterhouse divided by the number of birds sold to the slaughterhouse. The ‘FPLS’ (Foot-pad lesion score) variable of each flock is a weighted indicator based on the individual foot-pad lesion scores of a hundred birds sampled at the slaughterhouse, as required by the EU regulation^1^. Each bird is given a foot-pad lesion score: ‘0’ if no lesions were found, ‘1’ if the lesions were not too extensive and ‘2’ if they were. Then, the foot-pad lesion score of the flock is calculated by summing two products: 0.5 times the number of birds with a foot-pad lesion of grade ‘1’ and two times the number of birds with a foot-pad lesion of grade ‘2’.

#### Theme: economic indicator

Broiler producers use the European Production Efficiency Factor (EPEF) indicator to compare flocks. This indicator is calculated based on the others presented above: ‘mortality’, ‘FCR’, ‘mean age at slaughter’ and ‘mean weight at slaughter’. It’s the ‘mean weight at slaughter’ times the survival rate (a hundred minus the ‘mortality’) divided by the product of the ‘FCR’, ‘mean day of slaughter’ and a constant of 0.1 to adjust for units.

#### Theme: necropsy lesions

A list of 115 different necropsy lesions was available in the raw data. These lesions were grouped into nine anatomic systems or organs (e.g., cardiovascular system, spleen) and thirteen lesion types (e.g., fibrinous lesions, changes in the organ structure). Each group of lesions was turned into a binary variable coded as follows: ‘Y’ if at least one lesion from that group was observed in at least one of the five ‘screened flocks’, and ‘N’ if no lesion was observed in the ‘screened flocks’. The groups are described in detail in Additional file 2. Furthermore, for each observed lesion, pathologists assigned a confidence score based on their confidence that the lesion was present in the animal before collection or, rather, resulted in post-mortem deterioration of the animal. The scale was defined as follows: ‘high’ means pathologists were confident that the lesion existed antemortem, ‘low’ means pathologists had a low confidence that this lesion existed antemortem, ‘very Low’ means pathologists believed that the lesions did not exist antemortem and were rather due to post-mortem deterioration of the animal. Only the 37 lesions associated with a confidence score of ‘high’ and ‘low’ were retained for the analysis and are respectively named ‘high confidence score’ and ‘low confidence score’ necropsy lesions.

#### Theme: bacteria status

Nineteen variables related to the bacterial status of the flocks were built using 41 different bacteria taxa available in the raw data. These bacteria taxa could correspond to varying levels of classification of bacteria from the most detailed (ie, a bacteria species subtype) to the least (ie, bacteria genus). If a high classification level (such as genus) was used, all sub-levels were classified using the lowest common level (in this case, the genus). The following information was available for each ‘screened flock’: the observed bacteria taxa, the number of screened birds with samples from necropsy lesions where the bacteria grew, the observed bacteria growth intensity, and the sample’s localisation in which the bacteria were identified (see Additional file 2 for more information). In the case of ‘*Escherichia coli’ (E. coli) and* ‘*Clostridium perfringens’*, due to their ubiquity and the high probability of cross-contamination, only samples from internal organs (heart, liver, spleen and bone marrow) were kept for the analysis. Then, only when the growth intensity was over one in these samples was the result interpreted as the true presence of either bacteria. Finally, each variable was defined as a binary variable where ’Y’ means at least one of the bacteria from that taxa was identified in at least one of the birds of the ‘screened flock’, and ‘N’ means no bacteria taxa were found in the ‘screened flock’.

#### Theme: Eimeria status

Four variables related to the Eimeria status were defined based on the Eimeria species’ preferred tissue tropism in the intestines. Indeed, Eimeria species present tissue tropism, meaning that parasite localisation is usually associated with a specific Eimeria species: *E. acervulina* with the duodenal loop, *E. maxima* with the Meckel’s diverticulum, *E. tenella* with the cecum and *E. brunetti* with the rectum [23, 24]. In the raw data, each section of the intestines of each screened bird was given an infestation grade. This grade was defined at ‘0’ when no oocysts were found in the specific intestinal section, ‘1’ when 1 to 9 oocysts were observed, ‘2’ when 10 to 50 oocysts were observed, ‘3’ when more than 50 oocysts were observed. Each Emeria variable was turned into a categorical variable made of three levels defined according to the sum of the grade of Eimeria infestation across all five necropsied chickens: ‘no infestation’ if the sum equals 0, ‘low infestation’ if the sum is between one and three, ‘high infestation’ if the sum is above three.

#### Theme: viral circulation

One variable was created for each of the three endemic viruses considered important for poultry producers: infectious bronchitis virus (IBV), infectious bursal disease virus (IBDV), and avian metapneumovirus (aMPV). Virus circulation was estimated based on information available on vaccine type application, geometric mean titres of the serological samples, and RT-PCR results. Four categories for virus circulation were defined: ‘no’ if no virus circulation, ‘SUS’ if virus circulation is suspected, ‘HSUS’ if the virus circulation is highly suspected (category only defined to quantify IBV field circulation), and ‘yes’ if the virus circulation is confirmed. The definitions of these categories for each virus are available in Additional file 2.

### Data analyses

#### Data description

All data processing and calculations were performed using R (v.4.1.1) software [22]. The production performances and health characteristics of the ‘screened flocks’ were described, and outliers, uncorrelated variables and rare health events were identified. The correlation between quantitative variables was evaluated using Spearman correlation. The representativity of the ‘screened flocks’ compared to other flocks was assessed by comparing the mean production performances of the ‘screened flocks’ with the ones from the ‘recorded flocks’ using a two-tailed Welch’s two-sample t-test.

#### Multifactor analysis and hierarchical clustering

To describe the multivariate dataset, dimension reduction techniques followed by clustering were used. The dataset contains quantitative and qualitative variables regrouped into themes. Therefore, we implemented a multifactor analysis (MFA) to understand the main component defining the variability observed in the dataset, which is a dimension reduction technique used to explore the relationship between quantitative and/or qualitative mixed variables while balancing the influence of grouping [25]. The analysis was then followed by a hierarchical clustering analysis (HC). The HC is a means to visualise the variability and similarities between groups of individuals (i.e., flocks) regularly used in veterinary epidemiology [7, 12, 26–31]. Using MFA before HC allows the compilation of the Euclidean distance between groups, individuals, and variables and is used to reduce the number of variables to be included in the HC while mitigating the impact of multicollinearity [25]. The analyses were achieved through the FactoMineR package (v.2.11) [25]. For the MFA, the data had to be prepared based on its nature. First, all the continuous variables were centred and normalised. The mean was used to replace the missing quantitative data (only ‘FPLS’). In MFA, variables and individuals defined as ‘active’ are used to construct the high-dimensional Euclidean space, while ‘supplementary’ variables are only projected afterwards onto it and are independent. Supplementary individuals and variables were identified according to different criteria. First, outliers, defined as individuals (flocks) with very distinctive characteristics that contributed disproportionately to the first dimensions of the MFA compared to others, were set as supplementary individuals. Second, quantitative variables with no collinearity with other quantitative variables or calculated from other quantitative variables were kept as supplementary variables. Third, themes (context and information about aetiological agents) that could be used to interpret the dimensional space were defined as supplementary variables. All other variables were defined as active. To address the high-dimensionality (75 variables for 115 individuals) and low quality of the data, rare events were removed. These events were defined as any binary variables (necropsy lesions and bacteria) in which one of the categories encompassed fewer than 3% (3 or fewer individuals) of the records. Finally, the results of the MFA were described and more specifically, the variables’ contribution to the first two dimensions and their link to the supplementary qualitative variable based on an analysis of variance (F-test per variable and two-tailed Student’s t-test for the categories). The HC was then performed on the MFA projection. Clusters were identified from a dendrogram based on the Ward’s criterion to determine the optimal number of clusters and consolidated using the k-means algorithm [25]. Clustering is sensitive to outliers [32], to identify them, flocks creating a cluster of a single individual were set as a supplementary variable, and the analysis was rerun. To better describe the variability and similarities between the individuals, a two-tailed hypergeometric test for qualitative variables was used to identify the overrepresentation of variables in each cluster, and a two-tailed t-test was used to compare whether the mean of the quantitative variable of the cluster is equal to the overall mean [25]. The coherence and interpretability of the clusters across all the analyses were used as criteria to validate the definition of each cluster. Moreover, the impact of the assumptions made in the MFA was tested by exploring seven different scenarios. The results indicated that the clusters defined in the paper represented the most meaningful biological interpretation of the data. It should be noted that only the final results are described in this paper, but all other exploratory analyses are available in the R scripts attached to the paper (https://zenodo.org/records/18329566).

## Results

### Descriptive analysis

#### Screened flocks’ production and health, and welfare indicators and contextual qualitative descriptors

The final dataset used for the analysis (i.e., the 115 ‘screened flocks’) contained data collected between January 2022 and April 2023. The median of the number of chicken houses per farm was two (minimum = 1, maximum = 21). The median number of flocks screened per farm was two (minimum = 1, maximum = 5). Table 2 provides contextual information (i.e., farm type, region, season, ATB usage) about the ‘screened flocks’.

**Table 2:** Distribution of the contextual variables amongst the ‘screened flocks’ (n = 115)

Contextual variables	Number of flocks	% of flocks
Farm type		
1–2 chicken house	50	44%
>2 chicken houses	65	56%
NUTS1		
PL6	6	5%
PL8 South	25	22%
PL8 North	14	12%
PL9	70	61%
Season		
Autumn	21	18%
Spring	37	32%
Summer	32	28%
Winter	25	22%
ATB use		
Y	87	76%
N	28	24%

An overview of the production, health, and welfare indicators of the ‘screened flocks’ and ‘recorded flocks’ is provided in Table 3. Information on foot-pad lesions was missing in 6% of the ‘screened flocks’ (n = 7). The analysis allowed the identification of one flock with extreme values: very high ‘mortality’ of 40.69%, ‘FCR’ of 3.29, an ‘EPEF’ of 84.59 and a mean ‘age at slaughter’ of 42 days. No additional information from the dataset could explain the mortality rate observed in this specific flock, implying that it is due to an event unrelated to the health issues explored in the available data. This flock was therefore considered an outlier for the rest of the analysis.

**Table 3:** Description of the quantitative variables for the ‘screened flocks’ and ‘recorded flocks’

Performance or health and welfare indicators	Mean	SD	Median	Min	Max
Screened flocks (*n* = 115)					
EPEF	388.59	54.69	395.64	84.59	487.84
Mortality (%)**	6.010	5.430	5.080	0.620	40.690
DOA (%)	0.004	0.003	0.003	0.001	0.018
Condemnation (%)	0.590	0.500	0.470	0.130	3.440
Mean weight at slaughter (Kg)	2.520	0.180	2.540	1.700	2.990
Mean age at slaughter (Day)	38.910	1.410	38.880	35.000	42.030
FCR	1.590	0.180	1.570	1.290	3.190
FPLS* (*n* = 108)	64.720	37.090	61.000	0.000	152.000
Recorded flocks (*n* = 1 697)					
EPEF	403.68	51.56	405.52	84.59	771.57
Mortality (%)**	4.450	3.420	3.930	-18.150	40.690
DOA (%)	0.003	0.003	0.003	0.000	0.088
Condemnation (%)	0.570	0.350	0.480	0.050	3.440
Mean weight at slaughter (Kg)	2.570	0.160	2.580	1.700	3.000
Mean age at slaughter (Day)	39.100	1.610	39.000	30.000	46.000
FCR	1.570	0.130	1.570	0.810	3.190
FPLS* (*n* = 1 412)	61.900	40.630	56.000	0.000	200.000

For the tables’ column name: SD as standard deviation, max as the maximum, min as the minimum. For the variable names: EPEF is the European production efficiency factor, DOA is the death on arrival, FCR is the feed conversion ratio, and FPLS is the foot-pad lesion score

*FPLS are the only variables with missing information; the number of the flocks with available data is given in parentheses

**Mortality is a calculated value based on the number of chickens ordered at the hatchery and the final number of chickens sold at the slaughterhouse, meaning that the mortality could be negative due to specific unrecorded events. For example, this could occur during the delivery of additional chicks from the hatchery or the introduction of additional chickens by the farmer during the production cycle

**Fig. 2:**
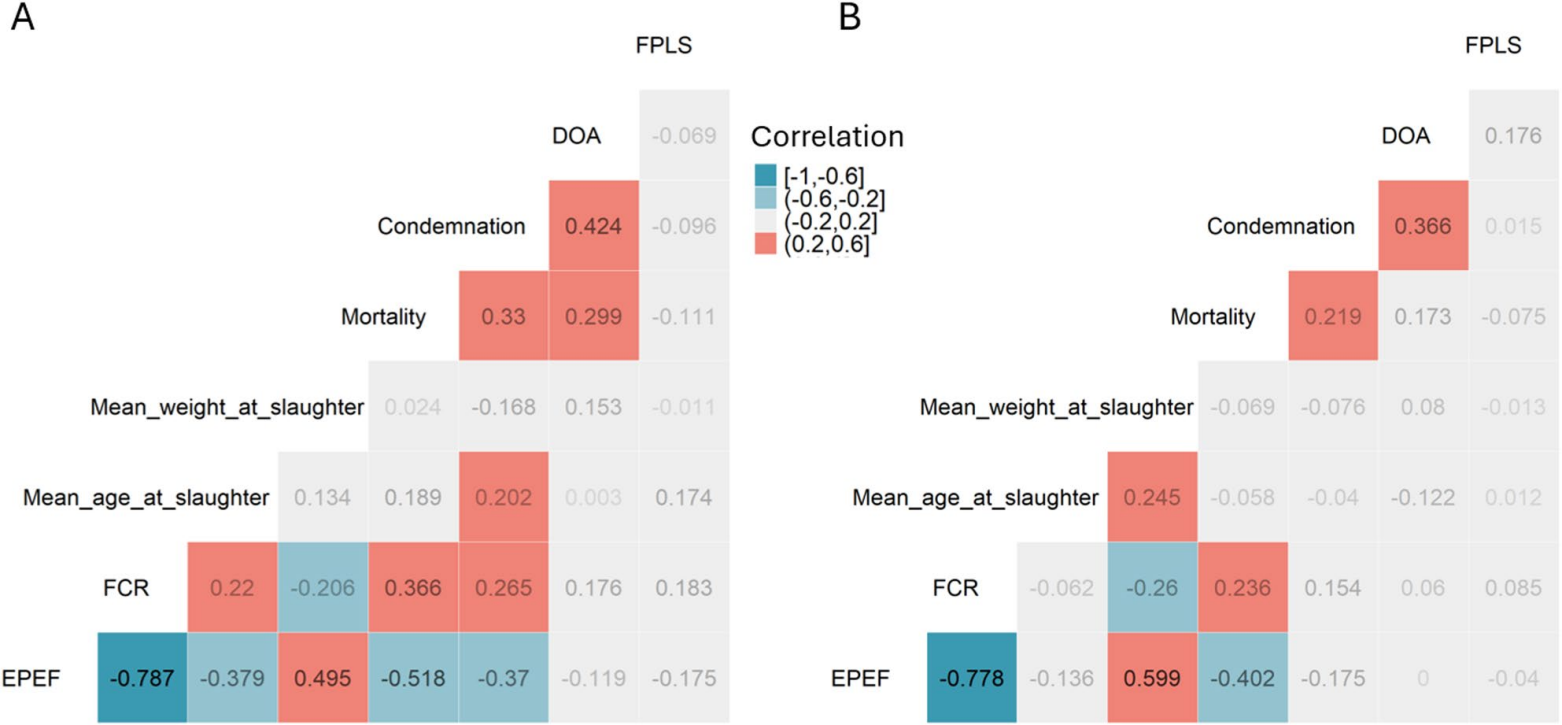
Figure **A** contains the screened flocks (n = 115); in Figure **B**, ‘recorded flocks’ were used for the analysis (n = 1697)

Correlation between quantitative variables is presented in Fig. 2.A. ‘EPEF’ was the single quantitative variable strongly correlated (higher than 0.5) with the others. Indeed, it is calculated from most other quantitative variables and was kept as a supplementary variable in the analysis. Amongst the remaining quantitative variables, the highest correlation was found between ‘DOA’ and ‘condemnation’ (|r| = 0.424; *p* < 0.001). All the other significant correlations (*p* < 0.05) identified between variables had a small correlation coefficient (|r| < 0.4 and |r| > 0.2). These variables were defined as active variables for the rest of the analysis. On the other hand, ‘FPLS’ (‘Foot-pad lesion scores’) was the only variable without any significant correlation with any of the other variables and, as such, was defined as a supplementary variable for the next step of the analysis.

The ‘screened flocks’, sampled from the ‘recorded flocks’, showed significantly lower performance and health compared to the rest of the study population. For the same ‘mean age at slaughter’, the ‘weight at the slaughter’ of the ‘screened flocks’ was lower (*p* < 0.001), while ‘mortality’ and ‘DOA’ were higher (*p* < 0.001 and *p* < 0.05, respectively). Furthermore, the dataset’s correlation matrices changed depending on the group of flocks considered, suggesting that the ‘screened flocks’ may not be representative of the ‘recorded flocks’ (see Fig. 2). Indeed, the correlation among the quantitative variables was generally weaker in the ‘recorded flocks’ compared to the ‘screened flocks’, with six comparisons losing significance (|r|< 0.2). However, a new correlation between age and weight at slaughter became significant (|r| = 0.245).

#### Laboratory qualitative data

An overview of the main screening results (‘Necropsy lesions’, ‘Bacteria status’, ‘Eimeria status’, and ‘Viral circulation’) is provided in Tables 4, 5, 6 and 7.

‘Necrosis or ulcer of the musculoskeletal system’ lesions, regrouping the common locomotor lesions ‘femoral head necrosis’ and ‘feet-pad dermatitis’, were observed in almost all the ‘screened flocks’ (*n* = 106, 92.2%). Moreover, the majority of the ‘screened flocks’ had at least one chicken presenting one of the high confidence score necropsy lesions: ‘uroliths in the ureters’ (*n* = 92, 80.0%), and ‘change in the composition (enlarged) of kidneys and/or ureters’ (n = 87, 75.7%). ‘Respiratory vascular congestion’ (hyperaemia or ecchymosis) was the only lesion associated with a low confidence score observed in most flocks (n = 89, 77.4%). Seventeen lesions were observed in less than 3% of the flocks. The corresponding variables were excluded from the analysis due to their scarcity.

**Table 4:** Frequency of necropsy lesions observed in the 115 ‘screened flocks’

System	Lesion type	Confidence score	Number of flocks	% of flocks
Musculo-skeletal	Necrosis - ulcer	High	106	92.2
Kidney and ureters	Urates/ Uroliths	High	92	80.0
Kidney and ureters	Change in composition	High	87	75.7
Spleen	Change in composition	High	48	41.7
Liver	Fibrin	High	30	26.1
Respiratory	Fibrin	High	30	26.1
Cardio-vascular	Fibrin	High	29	25.2
Celomic cavity	Fibrin	High	15	13.0
Liver	Change in composition	High	11	9.6
Musculo-skeletal	Vascular congestion	High	11	9.6
Gastrointestinal	Empty or abnormal content	High	10	8.7
Musculo-skeletal	Swelling or oedema	High	4	3.5
Respiratory	Vascular congestion	Low	89	77.4
Spleen	Vascular congestion	Low	54	47.0
Liver	Vascular congestion	Low	48	41.7
Kidney and ureters	Vascular congestion	Low	40	34.8
Cardio-vascular	Thinning, distended	Low	35	30.4
Respiratory	Swelling or oedema	Low	20	17.4
Cardio-vascular	Change in composition	Low	11	9.6
Cardio-vascular	Swelling or oedema	Low	8	7.0

Only lesions with a high or low confidence score are represented. Lesions associated with three or fewer flocks are also not presented, but available in the Additional file 3

Amongst the thirteen bacteria taxa identified in at least 4 ‘screened flocks’ (Table 5), ‘*E. coli’* was isolated in internal organs of one of the randomly selected flocks in almost two-thirds of the flocks (60.9%). The other bacteria taxa found in more than a fourth of the flocks were ‘*Enterococcus faecalis’* (29.6%), ‘Staphylococcus spp.’ (27.8%) and ‘*Clostridium perfringens’* (26.1%). In contrast, six rare bacteria taxa were identified in less than 3% of the flocks and were excluded from the dataset for the rest of the analysis.

**Table 5:** Frequency of bacteria taxa (species or genera) amongst the 115’ screened flocks’

Bacteria groups (species or genera) identified	Number of flocks	% of flocks
Escherichia coli*	70	60.9
*Enterococcus faecalis*	34	29.6
*Staphylococcus spp*	32	27.8
Clostridium perfringens*	30	26.1
*Enterococcus cecorum*	25	21.7
*Ornithobacterium rhinotracheale*	23	20.0
*Enterococcus faecium*	18	15.7
*Bordetella avium*	14	12.2
*Gallibacterium anatis*	11	9.6
*Riemerella anatipestifer*	8	7.0
*Bordetella hinzii*	7	6.1
*Klebsiella pneumoniae*	5	4.3
*Enterococcus hirae*	4	3.5

Bacteria taxa associated with three or fewer flocks are not presented, but available in the Additional file 3. The asterisk highlights that the presence of E.coli and Clostridium perfringens were defined a bit differently than for the other bacteria due to their ubiquity, see the methodology for further details

A majority of the ‘screened flocks’ (*n* = 75, 65.2%) had no sign of Eimeria infestation. The cecum preferred by ‘*E. tenella’* was the portion of the intestines most often colonised by oocysts (Table 6): 30.4% (*n* = 35) of the flocks presented evidence of colonisation in at least one chicken. In contrast, the duodenum preferred by ‘*E. acervulina’* was the section of the intestines that was the least colonised (n = 11, 9.6%).

**Table 6:** Frequency of Eimeria infestation grade amongst the 115 ‘screened flocks’

Parasite infestation	Number of flocks	% of flocks
Duodenum		
High	1	1%
Low	10	9%
No	104	90%
Meckel Diverticulum		
High	3	3%
Low	15	13%
No	97	84%
Cecum		
High	12	10%
Low	23	20%
No	80	70%
Rectum		
High	10	9%
Low	17	15%
No	88	77%

The estimated circulation of IBDV, IBV and aMPV viruses is available in Table 7. Almost half (42%, *n* = 48) of the sampled flocks showed evidence of field IBV virus circulation. Among those flocks, 70.8% were positive to the VAR 2 genotype (*n* = 34), 22.9% to the 793B genotype (*n* = 11),10.4% to the D274 genotype (*n* = 5), 4.2% to the Mass genotype (*n* = 2), 2.1% to the QX genotype (*n* = 1) and IB80 genotype (*n* = 1). All results are available in Additional file 3. The aMPV circulated in 20% of the flocks (*n* = 23) and IBDV in 2% (*n* = 2). However, virus genotypes for IBDV and aMPV were not explored further in the categories developed in Additional file 2.

**Table 7:** Frequency of virus circulation evidence amongst the 115’ screened flocks’

Virus circulation	Number of flocks	% of flocks
Avian metapneumovirus (aMPV)		
No	44	38%
SUS	48	42%
Yes	23	20%
Infectious bronchitis virus (IBV)		
No	40	35%
SUS	13	11%
HSUS	14	12%
Yes	48	42%
Infectious bursal disease virus (IBDV)		
No	75	65%
SUS	38	33%
Yes	2	2%

### MFA (Multifactor analysis)

 The MFA was performed on all 115 ‘screened flocks’ with the 53 variables available. Two quantitative variables (i.e., ‘FPLS’ and ‘EPEF’) were defined, respectively, as supplementary variables. A first attempt at HC on this data set created a cluster containing a single flock; this concerned the identified outlier and was therefore defined as supplementary. A second attempt at HC created again a cluster containing a single cluster. The newly identified flock had the highest condemnation rate (3.4%), the lowest mean weight at slaughter (1.70 kg) and the lowest mean age at slaughter (35 days), meaning that the production cycle was stopped early due to a specific event. As such, it was defined as an outlier and set as a supplementary individual. The final analysis was therefore done on 27 active variables and 113 active individuals.

**Fig. 3:**
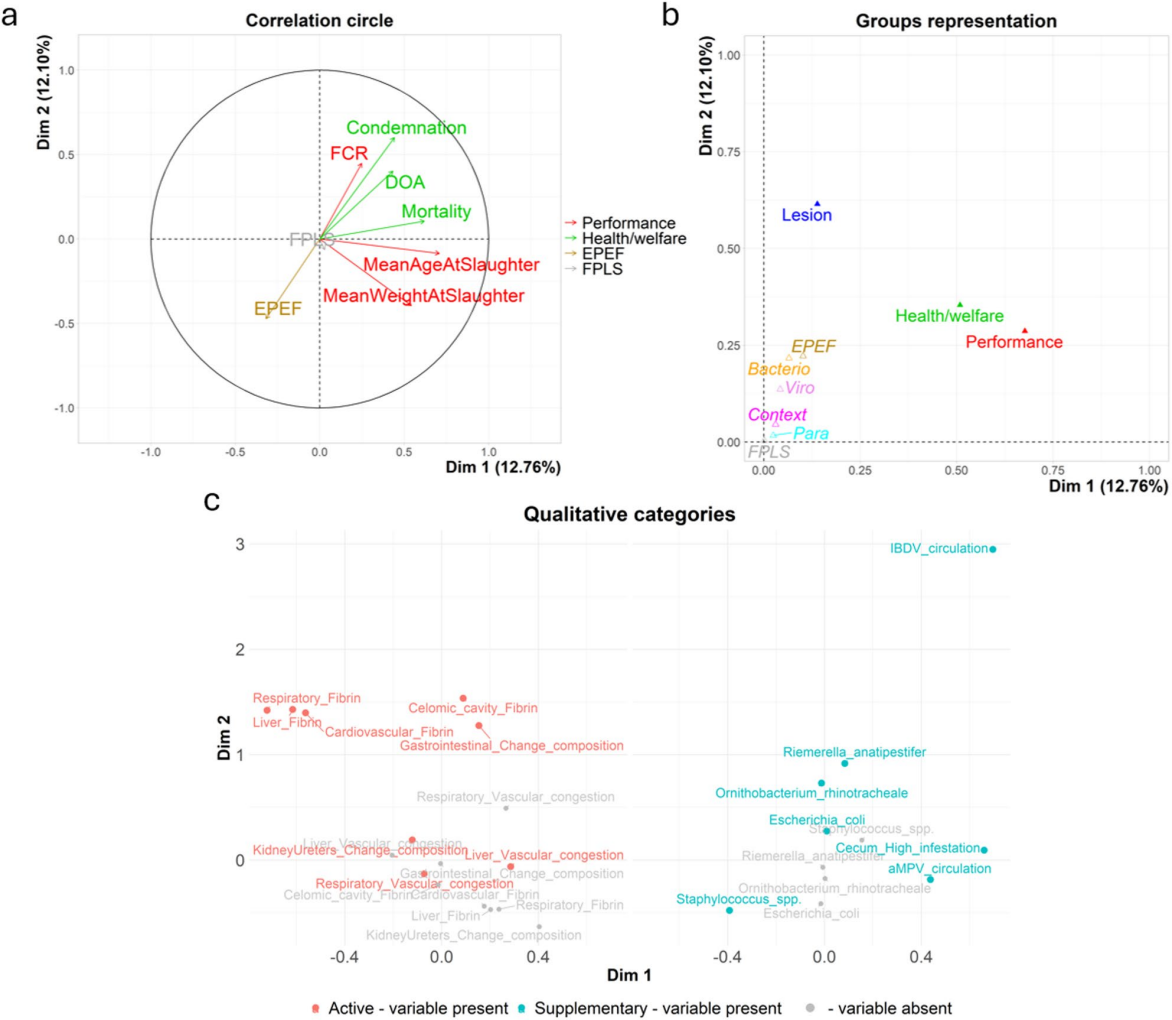
The correlation circle of the quantitative variable by themes (**a**), groups (theme) representation (**b**) and projection of the qualitative categories (supplementary in blue, active in red, categories link to an absence, meaning, lesions or bacteria not found in the flock, in grey) significantly associated with either the first or the second dimension (**c**)

**Fig. 4:**
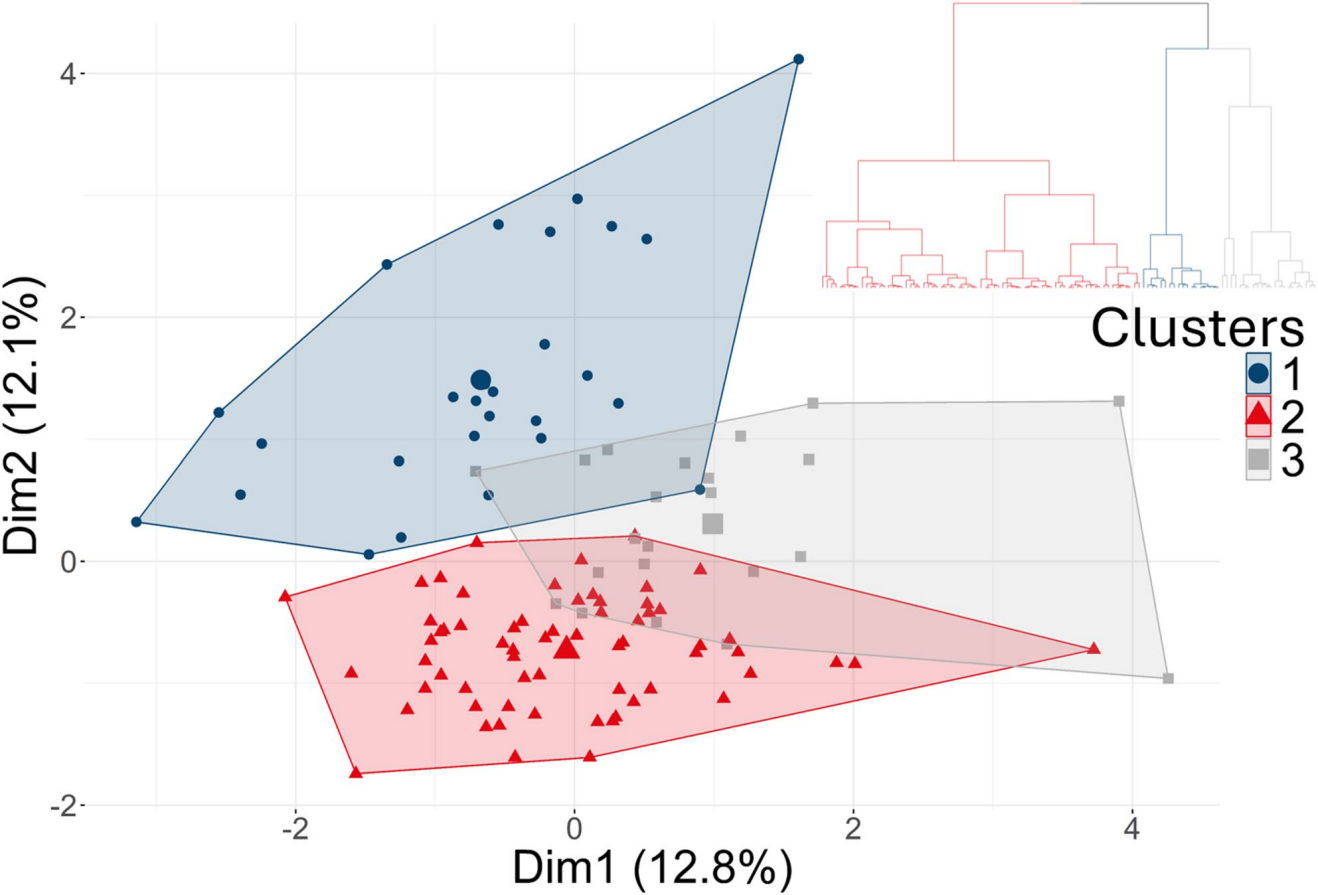
Flock projection in the MFA’s first two dimensions, coloured by clusters and hierarchical tree

The first 21 dimensions obtained with the MFA represented 95% of the dataset’s cumulative variability. The projection on the first two dimensions of the quantitative variables, the grouped variables and individuals are available in Figs. 3 and 4, respectively. These two dimensions reflect 24.8% of the whole dataset.

The correlation circle in Fig. 3a illustrates how the main quantitative variables contribute to the first two dimensions, while Fig. 3b provides a synthetic comparison of the groups of variables. Furthermore, the variance analysis allows us to identify the qualitative categories, projected in Fig. 3c, that best characterise the coordinates of the individuals on the first two dimensions. Amongst the 27 variables related to necropsy lesions used in the analysis, eight of them best characterised the individuals on the first two dimensions: ‘respiratory fibrin’, ‘liver fibrin’, ‘cardiovascular fibrin’, ‘liver vascular congestion’, ‘celomic cavity fibrin’, ‘kidney and ureters change in composition’, ‘respiratory vascular congestion’, ‘gastrointestinal change in composition. The contextual and aetiological variables were not used to construct the MFA and, therefore, are fully independent of the first two dimensions. However, ten of the 26 contextual and aetiological variables were correlated with the first two dimensions. These ten variables included four bacteria taxa (i.e., ‘*Staphylococcus spp*.’, ‘*Ornithobacterium rhinotracheale’*,* ‘E. coli’* and *‘Riemerella anatipestifer’*), two viral circulation variables (i.e., confirmed circulation of IBDV and aMPV), one Eimeria status (i.e. ‘High cecum infestation’) and two contextual variables (i.e., the flock’s region ‘PL9’ and ‘PL8a’).

### HC (Hierarchical clustering)

A partition in three clusters was inferred from the minimum ratio between two successive within-group inertias and is illustrated in Fig. 4. The clusters were named 1, 2 and 3 for ease, but a specific name was given based on the MFA active variables, which best characterised the group. All variables significantly characterising these groups are described in Tables 8, 9, 10 and 11 based on the characteristic of the theme they refer to (active, supplementary, qualitative or quantitative). The results, including the non-significant variables, are available in the Additional file 3.

**Table 8:** Mean and standard deviation (SD) for each quantitative variable per cluster

Variable	Overall		Cluster 1				Cluster 2				Cluster 3			
	Mean	SD	Mean	SD	*p*	V	Mean	SD	*p*	V	Mean	SD	*p*	V
Production performance														
FCR	1.6	0.1	1.6	0.1			1.5	0.1	***	-6.0	1.7	0.1	***	6.9
Mean age at slaughter	38.9	1.3	38.3	1.2	*	-2.6	38.9	1.4			39.7	0.9	**	2.9
Mean weight at slaughter	2.5	0.2	2.5	0.1	*	-2.2	2.6	0.1	**	3.0	2.5	0.2		
Health performance														
Mortality	5.7	4.4	5.1	3.0			4.5	2.4	***	-3.3	9.8	7.1	***	5.0
Condemnation	0.5	0.3	0.7	0.5	*	2.4	0.5	0.3	**	-3.1	0.6	0.3		
Supplementary														
EPEF	392.4	45.3	387.6	37.7			415.5	27.7	***	6.2	331.3	35.7	***	-7.1
FPLS	64.7	36.0	62.5	39.2			60.8	35.9			78.9	29.5	*	2.1

The variables came from quantitative themes set as active (‘Production performance’, ‘Health performance’) or supplementary (‘Economic indicator’) used in the MFA/HC and performed on 113 ‘screened flocks’ for each cluster. For each cluster and quantitative variable, the results of the t-test comparing the cluster to the overall mean are gathered in the table with V for the value of the t-test and p for the p-value. Only significant variables are described, and p-values are coded using asterisks: * for p < 0.05, ** for p < 0.01, *** for p < 0.001

**Table 9:** Frequency and proportion of each variable of the active qualitative theme ‘Context’ used in the MFA/HC performed on 113 flocks and for each cluster

Variable	Factor	Overall		Cluster 1				Cluster 2				Cluster 3			
		*N*	(%)	*N*	(%)	*p*	V	*N*	(%)	*p*	V	*N*	(%)	*p*	V
**Context**															
Region	PL6	6	5.3	0	0			5	7.9			1	4.2		
	PL8a	25	22.1	2	7.7	*	-2.1	18	28.6			5	20.8		
	PL8b	14	12.4	1	3.8			9	14.3			4	16.7		
	PL9	68	60.2	23	88.5	***	3.5	31	49.2	**	-2.6	14	58.3		
Farm type	Large	65	57.5	9	34.6	**	-2.6	37	58.7			19	79.2	*	2.4
	Small	48	42.5	17	65.4	**	2.6	26	41.3			5	20.8	*	-2.4
Season	Automn	21	18.6	5	19.2			15	23.8			1	4.2	*	-2.1
	Spring	36	31.9	6	23.1			20	31.7			10	41.7		
	Summer	31	27.4	9	34.6			15	23.8			7	29.2		
	Winter	25	22.1	6	23.1			13	20.6			6	25		

The variables are from the qualitative theme ‘Context’ used in the MFA/HC performed on 113 flocks. For each cluster and variable, the hypergeometric test results are gathered in the table with V for the sample estimate and p for the p-value. Only significant variables are described, and p-values are coded using asterisks: * for p < 0.05, ** for p < 0.01, *** for p < 0.001

**Table 10:** Frequency and proportion of each variable from the active qualitative theme ‘Necropsy lesions per cluster

Variable	Factor	Overall		Cluster 1				Cluster 2				Cluster 3			
		*N*	(%)	*N*	(%)	*p*	V	*N*	(%)	*p*	V	*N*	(%)	*p*	V
**Lesion (High confidence score)**															
Cardiovascular - Fibrin	No	86	76.1	2	7.4	***	-9.3	62	96.9	***	6.0	22	100	**	3.2
	Yes	27	23.9	25	92.6	***	9.3	2	3.1	***	-6.0	0	0	**	-3.2
Celomic cavity - Fibrin	No	98	86.7	17	63.0	***	-3.7	61	95.3	**	3.0	20	90.9		
	Yes	15	13.3	10	37.0	***	3.7	3	4.7	**	-3.0	2	9.1		
Kidney/ureters - Change in composition	No	26	23.0	3	11.1			21	32.8	**	2.8	2	9.1		
	Yes	87	77.0	24	88.9			43	67.2	**	-2.8	20	90.9		
Kidney/uretertes– Urates/ Uroliths	No	22	19.5	1	3.7	*	-2.5	13	20.3			8	36.4	*	2.1
	Yes	91	80.5	26	96.3	*	2.5	51	79.7			14	63.6	*	-2.1
Kidney/ureters - Vascular congestion	No	74	65.5	22	81.5	*	2.0	42	65.6			10	45.5	*	-2.1
	Yes	39	34.5	5	18.5	*	-2.0	22	34.4			12	54.5	*	2.1
Liver - Fib rin	No	85	75.2	0	0	***	-10.4	63	98.4	***	6.8	22	100	***	3.3
	Yes	28	24.8	27	100	***	10.4	1	1.6	***	-6.8	0	0	***	-3.3
Musculoskeletal - Necrosis, ulcer	No	9	8.0	2	7.4			2	3.1	*	-2.1	5	22.7	*	2.4
	Yes	104	92.0	25	92.6			62	96.9	*	2.1	17	77.3	*	-2.4
Respiratory - Fibrin	No	85	75.2	0	0	***	-10.4	63	98.4	***	6.8	22	100	***	3.3
	Yes	28	24.8	27	100	***	10.4	1	1.6	***	-6.8	0	0	***	-3.3
**Lesion (Low confidence score)**															
Cardiovascular - Thinning distended	No	79	69.9	23	85.2	*	2.0	44	68.8			12	54.5		
	Yes	34	30.1	4	14.8	*	-2.0	20	31.3			10	45.5		
Respiratory -Vascular congestion	No	24	21.2	10	37.0	*	2.2	12	18.8			2	9.1		
	Yes	89	78.8	17	63.0	*	-2.2	52	81.3			20	90.9		

The variables, from the qualitative theme ‘Necropsy lesions’ used in the MFA/HC performed on 113 flocks, are regrouped according to their confidence scores. For each cluster and variable, the hypergeometric test results are gathered in the table with V for the sample estimate and p for the p-value. Only significant variables are described, and p-values are coded using asterisks: * for p < 0.05, ** for p < 0.01, *** for p < 0.001

**Table 11:** Frequency and proportion of each variable of the supplementary qualitative theme per cluster

Variable	Factor	Overall		Cluster 1				Cluster 2				Cluster 3			
		*N*	(%)	*N*	(%)	*p*	V	*N*	(%)	*p*	V	*N*	(%)	*p*	V
**Bacteria status**															
* Bordetella avium*	No	100	88.5	26	96.3			53	82.8	*	-2.1	21	95.5	100	88.5
	Yes	13	11.5	1	3.7			11	17.2	*	2.1	1	4.5	13	11.5
Escherichia coli**	No	45	39.8	5	18.5	**	-2.6	31	48.4	*	2.1	9	40.9	45	39.8
	Yes	68	60.2	22	81.5	**	2.6	33	51.6	*	-2.1	13	59.1	68	60.2
* Staphylococcus spp*	No	81	71.7	21	77.8			41	64.1	*	-2.0	19	86.4	81	71.7
	Yes	32	28.3	6	22.2			23	35.9	*	2.0	3	13.6	32	28.3
**Eimeria status**															
Meckel’s diverticulum - (*E. maxima*)	High	3	2.7	0	0			2	3.1			1	4.5		
	Low	15	13.3	3	11.1			6	9.4			6	27.3		
	No	95	84.1	24	88.9			56	87.5			15	68.2	*	-2.1
Cecum *(E. tenella*)	High	12	10.6	1	3.7			5	7.8			6	27.3	*	2.5
	Low	23	20.4	7	25.9			10	15.6			6	27.3		
	No	78	69.0	19	70.4			49	76.6			10	45.5	*	-2.5
Rectum (*E. brunetti*)	High	10	8.8	0	0			7	10.9			3	13.6		
	Low	17	15.0	7	25.9			5	7.8	*	-2.4	5	22.7		
	No	86	76.1	20	74.1			52	81.3			14	63.6		
**Viral circulation**															
aMPV	No	44	38.9	16	59.3	*	2.4	24	37.5			4	18.2	*	-2.2
	SUS	46	40.7	9	33.3			26	40.6			11	50		
	Yes	23	20.4	2	7.4			14	21.9			7	31.8		

The qualitative supplementary themes used in the MFA/HC performed on 113 flocks are ‘Bacteria status’, ‘Eimeria status’, and ‘Virus circulation’. For each cluster and variable, the hypergeometric test results are gathered in the table with V for the value of the test and p for the p-value. Only significant variables are described, and p-values are coded using asterisks: * for p < 0.05, ** for p < 0.01, *** for p < 0.001

#### Cluster 2 – High-performing flocks

Cluster 2 included most of the ‘screened flocks’ (*n* = 64) and can be defined as a cluster of high-performing flocks associated with a low ‘FCR’, high ‘EPEF’, and high ‘weight at slaughter’ (see Table 8). These flocks also had better health performances, including fewer necropsy lesions. This was particularly true for fibrinous lesions in the liver, cardiovascular and respiratory tract, as well as for ‘change in composition (enlarged) in the kidneys or ureters’. However, ‘necrosis or ulcers of the musculoskeletal system’ were more common in this cluster than in the other. In terms of the presence of aetiological agent, Eimeria infestation, especially in the rectum, was less often observed, but two aetiological agents were over-represented, i.e., ‘*Bordetella avium’* and *‘Staphylococcus spp’* (Table 11).

#### Cluster 3 – Low-performing flocks

In opposition to cluster 2, cluster 3 (*n* = 22) can be defined as a cluster with flocks at an older age (longer production time) with low production and health performance raised in large farms (more than two chicken houses) (Table 8). Indeed, these flocks had a higher ‘FCR’, lower ‘EPEF’, and a higher ‘age at slaughter’, higher ‘mortality rate’ and higher ‘FPLS’ than the others. Particularly, one necrotic lesion was more frequent: ‘vascular congestion in the kidneys or ureters’, which has a high confidence score. Among the aetiological agents, the cluster showed evidence of infestation by Eimeria. Indeed, high infestations in the cecum or evidence of infestations in the Meckel’s diverticulum were observed more frequently in the cluster’s flocks. Furthermore, there were significantly more evidence of circulation (confirmed or suspected) of aMPV in this cluster than in the others.

#### Cluster 1 – Flocks with fibrinous lesions

Cluster 1 included 27 flocks and was characterised by birds slaughtered at a younger age and up to a lighter weight than the others. These flocks were generally raised on ‘small’ farms (less than two chicken houses), in the region ‘PL9’. They were more frequently associated with fibrinous lesions in the liver, cardiovascular, respiratory, and celomic cavity. ‘Uroliths were also observed more frequently in this cluster than in others. All these lesions were associated with a high confidence score, meaning they are likely antemortem lesions. In opposition to the two other clusters, this cluster presented no specificities regarding economic performance (‘EPEF’) but showed a higher condemnation rate. ‘*E. coli’ was the o*nly aetiological agent significantly more present in this cluster.

## Discussion

In our study, we investigated the relevance of endemic contagious diseases in Polish broiler flocks by exploring patterns in production and health performance variables, necropsy lesions, and the presence of aetiological pathogens. Indeed, the presence of an aetiological pathogen is not sufficient to understand the influence of a contagious endemic disease on production performances, especially when more than one pathogen circulates. The study population (‘screened flocks’) is part of the larger Polish broiler industry, which has greatly improved its efficiency in the past 30 years, and is the main European producer of poultry meat [33]. More specifically, these ‘screened flocks’ were produced just after the end of the COVID-19 pandemic during an avian flu epidemic, but in a period where low feed prices were prevalent, improving the Polish broiler production profitability [34]. In this context, the production performance of the ‘screened flocks’ was above the European average published by Van Limbergen et al. (2020), but lower than the Polish study case farm results presented by Adaszyńska-Skwirzyńska et al. (2025). Notably, they had an above-average cumulative mortality rate compared to both studies (6.01 ± 5.43 in the ‘screened flocks’, compared to the European mean of 3.82% ± 3.70). The ‘screened flocks’ were not associated with any major health issues to our knowledge (such as an avian flu outbreak), but were subject to high infectious pressure, as illustrated in our study by the diversity of pathogens found in these flocks: all three viruses from the screening assay (IBV, aMPV, IBDV) were detected in the study’s population (based on serology, vaccination history and PCR tests), as well as thirteen different bacteria and *Eimeria spp*. These aetiological agents could potentially explain the above-average mortality rates observed, especially as they were associated with a diversity of necropsy lesions in the birds (such as necrosis or ulcer of the musculoskeletal system’, ‘uroliths in the ureters’ or ‘change in the composition (enlarged) of kidneys and/or ureters’). To go beyond this general overview, our study investigated whether patterns across the ‘screened flocks’ in terms of production performance, health variables, and necropsy lesions could be observed in order to provide producers with a better understanding of their flocks’ health.

### Using typologies to understand how the screened flock’s performance and diseases are associated

Three flock types were identified using the HC analysis, each defined by specific characteristics in terms of production performance and presence of different pathogens.

#### High-performing flocks

The high-performing ‘screened flocks’ (cluster 2, *n* = 64) show better health and production performances (Table 8) compared with the overall ‘recorded flocks’ (Table 3), as well as with the European data published by Van Limbergen et al. (2020). Their production performances are close to Aviagen’s 2022 performance objectives [35], which are defined as the breed’s attainable standards, for a ‘mean age at slaughter’ of 39 days (i.e., FCR of 1.5 ± 0.1 and 1.47 and average ‘weight at slaughter’ of 2.6 ± 0.1 kg and 2.7 kg for the high-performing ‘screened flocks’ and Aviagen standards, respectively). These results highlight the strong production performance capacities of the studied farms. However, even if the cluster’s mean mortality (4.5% ± 2.4) was the lowest identified in our study, it remains higher than the reported European mean of 3.82% ± 3.70 [5], illustrating the potential for improvements in production performance. The reason for the relatively high mortality in these high-performing flocks is unclear, but it could indicate that the infectious pressure observed across all ‘screened flocks’ also affects them, despite their good production performance. However, issues arising from rapid growth or other environmental factors cannot be ruled out.

Two other characteristics defined those high-performing ‘screened flocks’. First, 96.9% of them presented ‘Musculoskeletal necrosis or ulcer’ lesions, the majority of which were necrosis of the femoral head (result not presented). According to other reports, these lesions could be associated with higher body weight and increased stocking density in birds [36, 37]. They are also considered indicators of broiler welfare issues in intensive production. These lesions, if spontaneous, have also been associated with ‘Staphylococcus spp.’ as a secondary infection [36, 38]. In the cluster, no information on their stocking density was available, but the ‘mean body weight at slaughter’ of the birds in the cluster was slightly but significantly higher than the ‘screened flocks’ (i.e., 2.6 kg and 2.5 kg, respectively). Also, the presence of ‘Staphylococcus spp.’ was overrepresented in this cluster. Based on the current understanding, the cluster characteristics could reflect this biological mechanism. Further investigation to determine if femoral head necrosis and secondary ‘*Staphylococcus spp’* infection are indeed correlated with increased stocking density and higher body weight in the Polish context is needed to better understand current production practices and their impact on welfare. Second, ‘*Bordetella avium’* was more frequently present in the flocks from this cluster than in the others. Biologically, this bacteria causes Turkey coryza, but its pathogenicity is considered opportunistic in broilers [39]. Its presence is not known to impact production performance. Hence, the presence of *Bordetella avium* in 11 of the high-performing flocks does not suggest any pathogenicity as well.

#### Low-performing flocks

The low-performing ‘screened flocks’ (cluster 3, *n* = 22) exhibit lower production and health performance, and were also associated with the presence of two aetiological agents commonly observed in these flocks. The first one, *Eimeria spp.*, has been reported to reduce a production performance indicator, to increase ‘FCR’ and increase ‘FPLS’ [40, 41], which are characteristics of this cluster. The observed pattern could therefore reflect a known biological phenomenon. However, estimating the impact of coccidiosis on production performance remains complex, especially in subclinical cases, as it is multifactorial, and co-infection with bacteria such as *Clostridium Perfringens* creates significant variation in production performance [42]. If causality between low production performance and coccidiosis cannot be assumed in this study, *Eimeria spp.* remains a major issue in broiler production, especially as pressure to reduce the use of coccidiostats (antimicrobial compounds) is growing [43, 44]. Unfortunately, the lack of information on coccidiosis management practices prevents this study from drawing any further conclusions.

The other more frequent aetiologic agent present in cluster 3 was aMPV. This observation was made taking into consideration serology and vaccine history, meaning that identification of vaccine strains is not impossible but limited. Biologically, over the past 10 years, the burden of this respiratory pathogen on European broiler flocks has been rising [45–47], while diverse aMPV subtype B strains are being reported across Europe, exhibiting greater diversity than the initial strains introduced in France in 1985 [46, 48]. Recent observations in Poland (Śmiałek, personal communication) suggest that aMPV infections have more significant health impacts at the end of the production, surpassing the effect observed with either IBV or IBDV. Our study shows an association between cluster 3, which is characterised by low production performances, and the presence of aMPV. Therefore, based on the observed pattern and external reports, the prevalence and current impact of aMPV in Polish broilers should be further investigated to determine whether the current management process for aMPV by farmers should be updated. In the meantime, vaccination remains the main aMPV control measure [45, 46].

#### Flocks with fibrinous lesions

The last type of flocks (cluster 1, *n* = 27) shows high frequencies of fibrinous lesions at necropsy, presence of ‘urolith’ and a higher condemnation rate, suggesting a probable sub-acute/chronic contagious disease affecting these ‘screened flocks’ at the end of production [49]. Despite the observed evidence of infection, these flocks did not perform worse than the average ‘screened flock’, especially on the economic ‘EPEF’ indicator. However, they were generally slaughtered at a smaller weight and a younger age. This could be due to the common practice of sending chickens to the slaughterhouse earlier than planned when clinical symptoms start to appear, to minimise potential losses. Furthermore, fewer treatments, especially antibiotics, are available for use due to the withdrawal period for human consumption, pushing producers to slaughter earlier to maximise their benefits instead of attempting treatment.

The only aetiological agent associated with this cluster was avian pathogenic *E. coli.* The necropsy results in the cluster’s flocks are also consistent with literature reports about colibacillosis, even if the lesions are not specific: perihepatitis, airsacculitis, pericarditis and peritonitis [50, 51]. Colibacillosis is also known as a major cause of carcass condemnation [51], which is consistent with the observed high rate of condemnation in the cluster. Furthermore, the infection can be primary, but is more often considered secondary [51, 52]. Our study did not identify other aetiological agents associated with the high prevalence of *E. coli.* Indeed, as sampling only occurred once at the end of production, this might have been too late for the primary causes of E. coli to be identified. Further work would be needed to study the potential primary source of infection or predisposing factors. To initiate the investigation, characteristics of the cluster can be utilised. For example, the ‘screened flocks’ in this cluster were most frequently associated with the region (PL9), which is characterised by a higher density of poultry production (Śmiałek, personal communication). The burden of a diverse set of endemic contagious diseases in highly dense areas has been shown to play a role [53]. Their single or grouped contribution to the performances of these flocks should be further investigated, especially as our study did not identify a specific aetiological agent more frequently present in these ‘screened flocks’. Moreover, environmental issues such as inadequate ventilation and poor hygiene practices have been identified as predisposing factors and should not be dismissed [52].

### Beyond the clusters

Four additional aetiological agents were associated with the first two dimensions of the MFA reflection, accounting for 24.8% of the dataset’s variability, and can provide further insight into the health and performance of the ‘screened flocks’.

#### Other aetiologic agents associated with specific performance and necropsy profiles

Among the aetiological agents identified as significant in the MFA, one virus (IBDV) and two bacteria taxa (‘*Ornithobacterium rhinotracheale’ and ‘Riemerella anatipestifer’*) do not appear in the clustering analysis, which can be used to draw further conclusions from our study.

All of those aetiological agents were pathogens of poultry flocks (i.e., ‘IBDV’, ‘*Ornithobacterium rhinotracheale’*, and ‘*Riemerella anatipestifer’*) associated with low production and health performances (see Fig. 3c). For example, the only two ‘screened flocks’ infected with IBDV despite vaccination exhibited low production and health performance, consistent with the immunosuppressive effects of IBDV infection [54]. This observation remains limited to two flocks, and to draw any conclusions, it would be necessary to know if more similar observations were made in other flocks. If the answer is yes, then it raises questions about the current vaccination protocol: are they properly carried out or could the vaccination failure be due to the presence of new IBDV strains circulating in Central Europe [55, 56]. Concerning flocks with ‘*Ornithobacterium rhinotracheale’*, they were characterised by the presence of fibrinous lesions and poor health performance (see Fig. 3c), which is expected for a respiratory pathogen observed in secondary infections in broilers [57]. Similarly, *Riemerella anatipestifer* is a respiratory pathogen that is scarcely described in broilers in the literature, but is more commonly described in duck or turkey flocks [58, 59]. In our study, we observed few cases of flocks infested with ‘*Riemerella anatipestifer’* (7%, 8 flocks). Furthermore, in the MFA, the bacterial taxa had close coordinates to ‘*Ornithobacterium rhinotracheale’*. Indeed, infection caused by both bacteria taxa in broilers can be misdiagnosed before laboratory testing as one another or as other aetiologic agents. *Riemerella anatipestifer*’s presence in flocks supports the need to investigate further the impact of this disease on Polish broiler production and raises questions about the route of infection, illustrating the importance of biosecurity and good hygiene practices in dense multi-species poultry production regions [59].

#### Prevalent pathogen and lesions not identified in the analysis

IBV was the virus with the most ‘screened flocks’ with evidence of circulation (after treatment of vaccine history, serology and RT-PCR results) among the ‘screened flocks’ (42%), but was not associated with any flock types or MFA dimensions. This is surprising because its health burden is reported to be one of the heaviest on the poultry industry, including in Poland [60, 61]. Hence, this observation could be due to the isolation of vaccine strains; however, the use of serology and vaccine information limits this hypothesis. On the other hand, Legnardi et al. (2019) investigated IBV circulation in Poland and reported the absence of systemic clinical signs in flocks despite evidence of virus circulation. They concluded that the widespread vaccination in Poland is effective in reducing the current IBV burden. Similar observations from Italy and France were reported [62–64]. This could explain why we did not observe a relation between the presence of IBV and our production and health variables, indicating that despite its wide presence in the population, the virus is not affecting health and production performance. Legnardi et al. (2019) also described a specificity concerning the Polish poultry industry, where a large number of IBV vaccination programs were being followed without any clear rationale. During this study, we observed similar practices: 25 different vaccination programs were used across the 115 ‘screened flocks’ from 59 different farms (results not shown). This practice increases the risk of vaccine virulence reversion and could play a role in the future emergence of new IBV strains [65]. Beyond the impact of IBV on flocks, our study re-highlights the need for timely contextual information to move towards a more rational approach towards IBV vaccination management.

### Challenges and limitations of data re-use

In this study, routinely collected data from farmers and veterinary laboratories were reused for research purposes, providing insight into the known health and production performance of broiler flocks in Poland. However, multiple biases are present in this data, highlighting that any generalisation of results to the entire study population (i.e., the ‘recorded flocks’) or to the overall Polish broiler population should be done with care. These biases include selection biases, missing farm-level data on known confounders, and information biases due to data management issues and laboratory limitations.

First, the flocks included in the analysis were not randomly selected, introducing selection bias as described. Our results confirmed that the production and health performance of ‘screened flocks’ were not representative of the entire study population (‘recorded flocks’). Producers may have preferentially selected flocks with poorer performances for screening. Observed health issue frequencies may therefore be overestimated in our sample due to the selection bias.

Second, data on farm management, nutrition and environmental factors were not available, which limited the extent of our analysis, as illustrated, for example, by the absence of information on coccidiosis management practices, stocking density, or production environmental status (such as ventilation and temperature).

Furthermore, it was the first time that these farms and the laboratory had made their data available to an external researcher for digital reuse at this scale. To do so, in the absence of a comprehensive automated data management process, they manually manipulated the data by integrating multiple spreadsheets or transcribing PDFs into spreadsheets. Such processes are known to create errors in the data, creating an information bias. To mitigate this issue for the transcribed data, any discrepancies identified were sent to the laboratory for correction and validation. The next steps of data integration and preparation for analysis were also automated to minimise further errors. Fully estimating the impact of such data errors is not possible.

Further limitations are present because the data were routinely collected for production purposes. First, the laboratory techniques used were defined to provide an operational screening for the farmer and their veterinarian, specific to their context, and not to be compared with other research results that answer specific research questions. For example, in studies examining the presence of IBV, techniques based on the complete sequencing of the S1 gene (the region where most of the IBV genetic variability is concentrated) are used, but these techniques are more expensive, more time-consuming and harder to implement [66]. Currently, they cannot be used for routine field screening by the Polish veterinary laboratory. Similarly, production performance data were created primarily to fulfil the industry’s needs, creating another information bias. For example, ‘mortality’ is calculated by farms as the number of chickens sold minus the number of chicks and is mainly used to calculate the flock’s economic benefit. A few ‘recorded flocks’ showed a negative ‘mortality’ (Table 3), illustrating the fact that this value is an estimation and not the true ‘cumulative mortality’. Indeed, it assumes that the no chicken movement occurred, which is indeed rare, and that the number of chicks ordered at the hatchery is the number delivered, which is often not true. Indeed, hatcheries can send additional chicks to anticipate any loss due to transport or other commercial reasons, which may underestimate mortality in our study. However, no abnormal mortality was observed in the ‘screened flocks’, especially considering that the observed mortality was high. This described information bias is therefore expected to have a low impact on our observations.

To address some of these limitations, undirected exploratory data analysis techniques (i.e., MFA and cluster analysis) were used because they require only a limited number of assumptions about the expected relationship in the data. However, they require assumptions based on the study’s objective. In this study, we assumed that the sampled data contained patterns that could provide information on contagious broiler diseases, based on flock health, production performance and necropsy findings. This is an assumption often made when defining flock health status [12, 31]. Furthermore, due to the relatively low quality of the data and its high dimensionality, it was assumed that outliers could be removed because they introduced more noise than variability, hiding patterns present in the data. To test the impact of these assumptions on our results, seven different scenarios were tested and compared. The results of these comparisons are not presented in the paper but are available in the attached R scripts. Briefly, regarding the outliers, their presence either created clusters of single individuals or, in some cases, even a chaining phenomenon [67]. Regarding the variables removed from the analysis, the supplementary data (‘rare bacteria’) were not assigned to any clusters. Regarding the necropsy lesions, their presence had little overall impact on the cluster results in all the scenarios tested. Still, it did sometimes further separate the individuals, creating a fourth cluster with less biological significance. All the flocks kept the same cluster assignment in the different scenarios investigated, except when the variable ‘FPLS’ was not considered as a supplementary variable. In that specific case, only 3 flocks (2.6%) changed their cluster of assignment, highlighting the low number of borderline flocks. These observations, in addition to the biological interpretation, confirm that the final results presented in the paper properly reflect the available data, despite the subjectivity of the methods.

### Untapped potential of data re-use

Reusing routinely collected data on animal health to enhance contextual knowledge of endemic contagious diseases has been promoted to increase coverage and timely data collection in ways that traditional data collection through research surveys cannot [7, 68]. However, we found only a few examples of similar work that integrated at least two different data sources and did not rely on an additional farm survey to fulfil the research goal [7, 13, 69, 70]. Despite the study’s limitations, the high-dimensional data enabled the formulation of multiple hypotheses to support future design studies, which could later be used to provide recommendations to producers. For example, based on the results, it could be recommended that more attention and care should be directed towards aMPV vaccination.

The study provides a first practical example of the value of routinely collected data reuse for health management by offering a multivariate description of flock health and generating animal health intelligence. In addition to univariate data description, network-based clustering techniques, similar to those used in this report and adapted to data streams, could be used to identify patterns, observe changes over time, and provide farmers with insightful multivariate information [14]. However, to be able to use these data for real-time decision-making, biases present in the data should be reduced, thereby requiring investment in improved data management and collection systems for the industry [9, 10, 17, 68]. This requirement impacts all stakeholders, including laboratories, as presented in this case study. However, building data management systems requires not only good data literacy but also knowledge of current data standardisation practices in the field, as multiple standards already exist. The gap in good data literacy can direct stakeholders toward the wrong digital choice for their needs, by, for example, not selecting the right technical service provider, who may not be aware of the specifics of the health data and their standards, creating later issues in the data management, but also in data re-use and sharing. Demonstrating the potential value of such systems is an essential first step when investing in integrated health data systems, as they are known to be costly, especially for small-scale farms, as in many cases in Poland [10, 21, 68]. However, supporting the industry in data literacy to improve digitalisation and the development of such tools is also essential for investment to be done properly.

## Conclusion

The study enabled the identification of three groups of flocks defined by specific performance status and necropsy lesions associated with known pathogens relevant to Polish broiler production, by reusing and integrating data regularly produced by the industry. As such, it demonstrates the value of this data in enhancing the monitoring and understanding of endemic contagious diseases and their interactions within a specific context. This study provides an example of how such data can be used to provide farmers and veterinarians with a deeper understanding of the primary characteristics and disease issues affecting their flocks, offering contextual information and hypotheses to enhance their disease prevention and management efforts.

## Supplementary Information

Additional file 1: Description of the laboratory method used to screen the broiler flocks.

Additional file 2: Description of the grouping or data integration rule used to define the variable used in the analysis. The file is organised in a workbook containing four worksheets. The first worksheet describes the workbook. The second worksheet contains the logic tree used for data integration to define the evidence of virus circulation in the ‘screened flocks’ using available information from the results of the ELISA tests, RT-PCR and provided vaccines. The third worksheet presents the grouping logic used to define the forty variables under the 'necropsy lesion' theme used in the analysis. The last worksheet presents the grouping logic used to define the 19 variables in the theme 'bacteria status' used in the study.

Additional file 3: Description of all results of the study, including variables with low, very low frequency (under 3%) and non-significant variables. The file is organised in a workbook containing six worksheets. The first worksheet describes the workbook. The following three worksheets present the frequency of necropsy lesions, bacterial taxa, and IBV strain circulation amongst the 115 ‘screened flocks’. The remaining two worksheets present the results of the hierarchical cluster analysis for the quantitative variables first, followed by the qualitative variables.

## Data Availability

The data analysed during the current study are available in the Zenodo repository, [10.5281/zenodo.17406208](10.5281/zenodo.17406208).

## Footnotes

1 REGULATION (EU) 2017/625 OF THE EUROPEAN PARLIAMENT AND OF THE COUNCIL of 15 March 2017.

## References

[1] FAO. World Agriculture towards 2030/2050: the 2012 revision. 2012. https://www.fao.org/3/ap106e/ap106e.pdf. Accessed 18 Apr 2024.

[2] Oviedo-Rondón EO. Optimizing the health of broilers. Burleigh Dodds Ser Agric Sci. 2022. 10.19103/AS.2022.0104.11.

[3] Franzo G, Legnardi M, Faustini G, Tucciarone CM, Cecchinato M. When Everything Becomes Bigger: Big Data for Big Poultry Production. Animals. 2023;13:1804. 10.3390/ani13111804. 10.3390/ani13111804 PMC10252109 37889739

[4] Gržinić G, Piotrowicz-Cieślak A, Klimkowicz-Pawlas A, Górny RL, Ławniczek-Wałczyk A, Piechowicz L, et al. Intensive poultry farming: A review of the impact on the environment and human health. Sci Total Environ. 2023;858:160014. 10.1016/j.scitotenv.2022.160014. 36368402 10.1016/j.scitotenv.2022.160014

[5] Van Limbergen T, Sarrazin S, Chantziaras I, Dewulf J, Ducatelle R, Kyriazakis I, et al. Risk factors for poor health and performance in European broiler production systems. BMC Vet Res. 2020;16:287. 10.1186/s12917-020-02484-3. 32787841 10.1186/s12917-020-02484-3 PMC7425143

[6] Jones PJ, Niemi J, Christensen J-P, Tranter RB, Bennett RM. A review of the financial impact of production diseases in poultry production systems. Anim Prod Sci. 2019;59:1585. 10.1071/AN18281.

[7] Pandolfi F, Edwards SA, Maes D, Kyriazakis I. Connecting different data sources to assess the interconnections between Biosecurity, Health, Welfare, and performance in commercial pig farms in great Britain. Front Vet Sci. 2018;5. 10.3389/fvets.2018.00041. 10.3389/fvets.2018.00041 PMC5845643 29560358

[8] Hepworth PJ, Nefedov AV, Muchnik IB, Morgan KL. Broiler chickens can benefit from machine learning: support vector machine analysis of observational epidemiological data. J R Soc Interface. 2012;9:1934–42. 10.1098/rsif.2011.0852. 22319115 10.1098/rsif.2011.0852 PMC3385756

[9] Bumanis N, Arhipova I, Paura L, Vitols G, Jankovska L. Data conceptual model for smart poultry farm management system. Procedia Comput Sci. 2022;200:517–26. 10.1016/j.procs.2022.01.249.

[10] Olejnik K, Popiela E, Opaliński S. Emerging precision management methods in poultry sector. Agriculture. 2022;12:718. 10.3390/agriculture12050718.

[11] Rushton J, Huntington B, Gilbert W, Herrero M, Torgerson PR, Shaw APM, et al. Roll-out of the global burden of animal diseases programme. Lancet. 2021;397:1045–6. 10.1016/S0140-6736(21)00189-6. 33549170 10.1016/S0140-6736(21)00189-6

[12] Buzdugan SN, Alarcon P, Huntington B, Rushton J, Blake DP, Guitian J. Enhancing the value of meat inspection records for broiler health and welfare surveillance: longitudinal detection of relational patterns. BMC Vet Res. 2021;17:278. 10.1186/s12917-021-02970-2. 34407823 10.1186/s12917-021-02970-2 PMC8371771

[13] Hibbard R, Fourtune L, Pinson M, Delpont M, Vaillancourt J-P, Faverjon C, et al. Moving beyond metrics: capturing the clinical context behind antibiotic prescriptions in French broiler production. Prev Vet Med. 2026;246:106700. 10.1016/j.prevetmed.2025.106700.

[14] Atif M, Farooq M, Shafiq M, Alballa T, Abdualziz Alhabeeb S, Abd El-Wahed Khalifa H. Uncovering the impact of outliers on clusters’ evolution in Temporal data-sets: an empirical analysis. Sci Rep. 2024;14:30674. 10.1038/s41598-024-75928-7. 39730370 10.1038/s41598-024-75928-7 PMC11681016

[15] Swirski AL, Kasab-Bachi H, Rivers J, Wilson JB. Data driven enhancements to the intestinal integrity (I2) index: A novel approach to support poultry sustainability. Agriculture. 2020;10:320. 10.3390/agriculture10080320.

[16] Doidge C, Ånestad LM, Burrell A, Frössling J, Palczynski L, Pardon B, et al. A living lab approach to Understanding dairy farmers’ technology and data needs to improve herd health: focus groups from 6 European countries. J Dairy Sci. 2024;107:5754–78. 10.3168/jds.2024-24155. 38490555 10.3168/jds.2024-24155

[17] Rojo-Gimeno C, van der Voort M, Niemi JK, Lauwers L, Kristensen AR, Wauters E. Assessment of the value of information of precision livestock farming: A conceptual framework. NJAS - Wagening J Life Sci. 2019;90–1. 10.1016/j.njas.2019.100311.

[18] Top J, Janssen S, Boogaard H, Knapen R, Şimşek-Şenel G. Cultivating FAIR principles for agri-food data. Comput Electron Agric. 2022. 10.1016/j.compag.2022.106909.

[19] Eurostat. Production of meat: poultry. 2024. 10.2908/TAG00043.

[20] Kowalska A, Olszańska A, Szymańska J, Paskudzka K. International competitiveness of the poultry sector in Poland over the last 20 years. Rocz Nauk Stowarzyszenia Ekon Rol Agrobiznesu. 2023;24. 10.5604/01.3001.0053.9504.

[21] Zielińska-Chmielewska A, Kaźmierczyk J, Jaźwiński I. Quantitative research on profitability measures in the Polish meat and poultry industries. Agronomy. 2022;12:92. 10.3390/agronomy12010092.

[22] R Core Team. R: A Language and environment for statistical computing. Vienna, Austria.: R Foundation for Statistical Computing; 2024.

[23] Mesa-Pineda C, Navarro-Ruíz JL, López-Osorio S, Chaparro-Gutiérrez JJ, Gómez-Osorio LM. Chicken coccidiosis: from the parasite lifecycle to control of the disease. Front Vet Sci. 2021;8:787653. 10.3389/fvets.2021.787653. 34993246 10.3389/fvets.2021.787653 PMC8724208

[24] Shivaramaiah C, Barta JR, Hernandez-Velasco X, Téllez G, Hargis BM. Coccidiosis: recent advancements in the immunobiology of Eimeria species, preventive measures, and the importance of vaccination as a control tool against these apicomplexan parasites. Vet Med Res Rep. 2014;5:23–34. 10.2147/VMRR.S57839. 10.2147/VMRR.S57839 PMC7337151 32670843

[25] Husson F, Josse J, Pages J. Principal component methods - hierarchical clustering - partitional clustering: why would we need to choose for visualizing data? 2010. http://factominer.free.fr/more/HCPC_husson_josse.pdf

[26] Bâtie C, Ha LTT, Loire E, Truong DB, Tuan HM, Cuc NTK, et al. Characterisation of chicken farms in vietnam: A typology of antimicrobial use among different production systems. Prev Vet Med. 2022;208:105731. 10.1016/j.prevetmed.2022.105731. 36027681 10.1016/j.prevetmed.2022.105731

[27] Poupaud M, Goutard FL, Phouthana V, Muñoz Viera F, Caro D, Patriarchi A, et al. Different kettles of fish: varying patterns of antibiotic use on pig, chicken and fish farms in Lao PDR and implications for antimicrobial resistance strategies. Transbound Emerg Dis. 2022;69:3940–51. 10.1111/tbed.14766. 36401809 10.1111/tbed.14766 PMC10108286

[28] Delpont M, Guinat C, Guérin J-L, Le leu E, Vaillancourt J-P, Paul MC. Biosecurity measures in French poultry farms are associated with farm type and location. Prev Vet Med. 2021;195:105466. 10.1016/j.prevetmed.2021.105466. 34419776 10.1016/j.prevetmed.2021.105466

[29] Alvarez S, Timler CJ, Michalscheck M, Paas W, Descheemaeker K, Tittonell P, et al. Capturing farm diversity with hypothesis-based typologies: an innovative methodological framework for farming system typology development. PLoS ONE. 2018;13:e0194757. 10.1371/journal.pone.0194757. 29763422 10.1371/journal.pone.0194757 PMC5953459

[30] Ornelas-Eusebio E, García-Espinosa G, Laroucau K, Zanella G. Characterization of commercial poultry farms in mexico: towards a better Understanding of biosecurity practices and antibiotic usage patterns. PLoS ONE. 2020;15:e0242354. 10.1371/journal.pone.0242354. 33259478 10.1371/journal.pone.0242354 PMC7707464

[31] Dupuy C, Morignat E, Maugey X, Vinard J-L, Hendrikx P, Ducrot C, et al. Defining syndromes using cattle meat inspection data for syndromic surveillance purposes: a statistical approach with the 2005–2010 data from ten French slaughterhouses. BMC Vet Res. 2013;9:88. 10.1186/1746-6148-9-88. 23628140 10.1186/1746-6148-9-88 PMC3681570

[32] Wani AA. Comprehensive analysis of clustering algorithms: exploring limitations and innovative solutions. PeerJ Comput Sci. 2024;10:e2286. 10.7717/peerj-cs.2286. 39314716 10.7717/peerj-cs.2286 PMC11419652

[33] Utnik-Banaś K, Żmija J, Krawczyk J, Połtowicz K. Changes in technical efficiency of the broiler production in Poland, 1994–2013. Br Poult Sci. 2018;59:245–9. 10.1080/00071668.2017.1417541. 29235892 10.1080/00071668.2017.1417541

[34] Adaszyńska-Skwirzyńska M, Konieczka P, Bucław M, Majewska D, Pietruszka A, Zych S, et al. Analysis of the production and economic indicators of broiler chicken rearing in 2020–2023: A case study of a Polish farm. Agriculture. 2025;15:139. 10.3390/agriculture15020139.

[35] Aviagen. Ross 308/ 308 FF Broiler: Performance Objectives.pdf. 0822-AVNR-157. 2022. https://aviagen.com/assets/Tech_Center/Ross_Broiler/RossxRoss308-BroilerPerformanceObjectives2022-EN.pdf. Accessed 3 Apr 2024.

[36] Ge H, Yu Y, Zhang Y, Zhou Z. Changes of bone and articular cartilage in broilers with femoral head necrosis. Poult Sci. 2024;103:104127. 10.1016/j.psj.2024.104127. 39111237 10.1016/j.psj.2024.104127 PMC11343062

[37] van der Eijk JAJ, van Harn J, Gunnink H, Melis S, van Riel JW, de Jong IC. Fast- and slower-growing broilers respond similarly to a reduction in stocking density with regard to gait, Hock burn, skin lesions, cleanliness, and performance. Poult Sci. 2023;102:102603. 10.1016/j.psj.2023.102603. 36996512 10.1016/j.psj.2023.102603 PMC10070940

[38] Aarestrup FM, Agersø Y, Ahrens P, Jørgensen JCØ, Madsen M, Jensen LB. Antimicrobial susceptibility and presence of resistance genes in Staphylococci from poultry. Vet Microbiol. 2000;74:353–64. 10.1016/S0378-1135(00)00197-8. 10831857 10.1016/s0378-1135(00)00197-8

[39] Nielsen SS, Bicout DJ, Calistri P, Canali E, Drewe JA, Garin-Bastuji B, et al. Assessment of animal diseases caused by bacteria resistant to antimicrobials: poultry. EFSA J. 2021;19:e07114. 10.2903/j.efsa.2021.7114. 34987629 10.2903/j.efsa.2021.7114 PMC8703241

[40] Choi J, Goo D, Sharma MK, Ko H, Liu G, Paneru D, et al. Effects of different Eimeria inoculation doses on growth Performance, daily feed Intake, gut Health, gut Microbiota, foot pad Dermatitis, and Eimeria gene expression in broilers Raised in floor pens for 35 days. Animals. 2023;13:2237. 10.3390/ani13132237. 37444035 10.3390/ani13132237 PMC10339913

[41] Parker CD, Lister SA, Gittins J. Impact assessment of the reduction or removal of ionophores used for controlling coccidiosis in the UK broiler industry. Vet Rec. 2021;189:e513. 10.1002/vetr.513. 34101192 10.1002/vetr.513

[42] Nicholds JF, McQuain C, Hofacre CL, Mathis GF, Fuller AL, Telg BE, et al. The effect of different species of Eimeria with clostridium perfringens on performance parameters and induction of clinical necrotic enteritis in broiler chickens. Avian Dis. 2020;65:132–7. 10.1637/aviandiseases-D-20-00106. 10.1637/aviandiseases-D-20-00106 34339132

[43] FVE. FVE position paper on coccidia control in poultry. 2022. https://fve.org/cms/wp-content/uploads/001_FVE-coccidia-control-2022_adopted.pdf. Accessed 4 Aug 2025.

[44] Gilbert W, Bellet C, Blake DP, Tomley FM, Rushton J. Revisiting the economic impacts of Eimeria and its control in European intensive broiler systems with a recursive modeling approach. Front Vet Sci. 2020;7. 10.3389/fvets.2020.558182. 10.3389/fvets.2020.558182 PMC7674784 33251254

[45] Tucciarone CM, Franzo G, Lupini C, Alejo CT, Listorti V, Mescolini G, et al. *Avian metapneumovirus* circulation in Italian broiler farms. Poult Sci. 2018;97:503–9. 10.3382/ps/pex350. 29253264 10.3382/ps/pex350

[46] Lupini C, Tucciarone CM, Mescolini G, Quaglia G, Graziosi G, Turblin V, et al. Longitudinal survey on aMPV circulation in French broiler flocks following different vaccination strategies. Animals. 2023;13:57. 10.3390/ani13010057. 10.3390/ani13010057 PMC9817960 36611670

[47] Tucciarone CM, Legnardi M, Cecchinato M, Franzo G, Poletto F, Miccio L, et al. Research note: indirect evidence of avian metapneumovirus circulation in broilers in Italy. Poult Sci. 2024;103:104182. 10.1016/j.psj.2024.104182. 39154613 10.1016/j.psj.2024.104182 PMC11471103

[48] Franzo G, Legnardi M, Mescolini G, Tucciarone CM, Lupini C, Quaglia G, et al. Avian metapneumovirus subtype B around europe: a phylodynamic reconstruction. Vet Res. 2020;51:88. 10.1186/s13567-020-00817-6. 32641149 10.1186/s13567-020-00817-6 PMC7346485

[49] Luyendyk JP, Schoenecker JG, Flick MJ. The multifaceted role of fibrinogen in tissue injury and inflammation. Blood. 2019;133:511–20. 10.1182/blood-2018-07-818211. 30523120 10.1182/blood-2018-07-818211 PMC6367649

[50] Dziva F, Stevens MP. Colibacillosis in poultry: unravelling the molecular basis of virulence of avian pathogenic Escherichia coli in their natural hosts. Avian Pathol. 2008;37:355–66. 10.1080/03079450802216652. 18622850 10.1080/03079450802216652

[51] Kromann S, Baig S, Stegger M, Olsen RH, Bojesen AM, Jensen HE, et al. Longitudinal study on background lesions in broiler breeder flocks and their progeny, and genomic characterisation of Escherichia coli. Vet Res. 2022;53:52. 10.1186/s13567-022-01064-7. 35799204 10.1186/s13567-022-01064-7 PMC9264609

[52] Salles GBC, Pilati GVT, Savi BP, Muniz EC, Dahmer M, Vogt JR, et al. Surveillance of avian metapneumovirus in Non-Vaccinated chickens and Co-Infection with avian pathogenic Escherichia coli. Microorganisms. 2024;12:56. 10.3390/microorganisms12010056. 10.3390/microorganisms12010056 PMC10820577 38257889

[53] Franzo G, Tucciarone CM, Moreno A, Legnardi M, Massi P, Tosi G, et al. Phylodynamic analysis and evaluation of the balance between anthropic and environmental factors affecting IBV spreading among Italian poultry farms. Sci Rep. 2020;10:7289. 10.1038/s41598-020-64477-4. 32350378 10.1038/s41598-020-64477-4 PMC7190837

[54] Franciosini MP, Davidson I. A walk through Gumboro disease. Poultry. 2022;1:229–42. 10.3390/poultry1040020.

[55] Lupini C, Felice V, Silveira F, Mescolini G, Berto G, Listorti V, et al. Comparative in vivo pathogenicity study of an ITA genotype isolate (G6) of infectious bursal disease virus. Transbound Emerg Dis. 2020;67:1025–31. 10.1111/tbed.13421. 31715072 10.1111/tbed.13421

[56] Mató T, Medveczki A, Kiss I. Research note: hidden infectious bursal disease virus infections in central Europe. Poult Sci. 2022;101:101958. 10.1016/j.psj.2022.101958. 35691238 10.1016/j.psj.2022.101958 PMC9194827

[57] Barbosa EV, Cardoso CV, Silva R, de CF, Cerqueira A, de MF, Liberal MHT, Castro HC. Ornithobacterium rhinotracheale: an update review about an emerging poultry pathogen. Vet Sci. 2019;7:3. 10.3390/vetsci7010003. 31892160 10.3390/vetsci7010003 PMC7157751

[58] Nowaczek A, Dec M, Stępień-Pyśniak D, Wilczyński J, Urban-Chmiel R. Characterization of Riemerella anatipestifer strains isolated from various poultry species in Poland. Antibiotics. 2023;12:1648. 10.3390/antibiotics12121648. 38136682 10.3390/antibiotics12121648 PMC10740677

[59] Tzora A, Skoufos S, Bonos E, Fotou K, Karamoutsios A, Nelli A, et al. Identification by MALDI-TOF MS and antibiotic resistance of Riemerella anatipestifer, isolated from a clinical case in commercial broiler chickens. Vet Sci. 2021;8:29. 10.3390/vetsci8020029. 33671477 10.3390/vetsci8020029 PMC7922512

[60] de Wit JJ, Cazaban C, Dijkman R, Ramon G, Gardin Y. Detection of different genotypes of infectious bronchitis virus and of infectious bursal disease virus in European broilers during an epidemiological study in 2013 and the consequences for the diagnostic approach. Avian Pathol. 2018;47:140–51. 10.1080/03079457.2017.1387231. 28972403 10.1080/03079457.2017.1387231

[61] De Wit JJ (Sjaak), Cook JKA, editors. Spotlight on avian coronaviruses. Avian Pathol. 2020;49:313–6. 10.1080/03079457.2020.1761010 10.1080/03079457.2020.1761010 32374218

[62] Franzo G, Tucciarone CM, Blanco A, Nofrarías M, Biarnés M, Cortey M, et al. Effect of different vaccination strategies on IBV QX population dynamics and clinical outbreaks. Vaccine. 2016;34:5670–6. 10.1016/j.vaccine.2016.09.014. 27670071 10.1016/j.vaccine.2016.09.014 PMC7173296

[63] de Wit JJ, de Wit MK, Cook JKA. Infectious bronchitis virus types affecting European Countries—A review. Avian Dis. 2021;65:643–8. 10.1637/aviandiseases-D-21-00106. 35068110 10.1637/aviandiseases-D-21-00106

[64] Franzo G, Faustini G, Tucciarone CM, Poletto F, Tonellato F, Cecchinato M, et al. The effect of global Spread, Epidemiology, and control strategies on the evolution of the GI-19 lineage of infectious bronchitis virus. Viruses. 2024;16:481. 10.3390/v16030481. 38543846 10.3390/v16030481 PMC10974917

[65] Legnardi M, Franzo G, Koutoulis KC, Wiśniewski M, Catelli E, Tucciarone CM, et al. Vaccine or field strains: the Jigsaw pattern of infectious bronchitis virus molecular epidemiology in Poland. Poult Sci. 2019;98:6388–92. 10.3382/ps/pez473. 31399745 10.3382/ps/pez473 PMC6870560

[66] Legnardi M, Tucciarone CM, Franzo G, Cecchinato M. Infectious bronchitis virus Evolution, diagnosis and control. Vet Sci. 2020;7:79. 10.3390/vetsci7020079. 32580381 10.3390/vetsci7020079 PMC7356646

[67] Klutchnikoff N, Poterie A, Rouvière L. Statistical analysis of a hierarchical clustering algorithm with outliers. J Multivar Anal. 2022;192:105075. 10.1016/j.jmva.2022.105075.

[68] Vial F, Tedder A. Tapping the Vast Potential of the Data Deluge in Small-scale Food-Animal Production Businesses: Challenges to Near Real-time Data Analysis and Interpretation. Front Vet Sci. 2017;4. 10.3389/fvets.2017.00120. 10.3389/fvets.2017.00120 PMC5592208 28932740

[69] Junghans A, Deseniß L, Louton H. Data evaluation of broiler chicken rearing and slaughter—An exploratory study. Front Vet Sci. 2022;9. 10.3389/fvets.2022.957786. 10.3389/fvets.2022.957786 PMC9583157 36277067

[70] Zühlke I, Berezowski J, Bodmer M, Küker S, Göhring A, Rinaldi F, et al. Factors associated with cattle necropsy submissions in Switzerland, and their importance for surveillance. Prev Vet Med. 2021;187:105235. 10.1016/j.prevetmed.2020.105235. 33453476 10.1016/j.prevetmed.2020.105235

