# Supplementary material for: Integrating heterogeneous data to address endemic diseases in broiler production: insights from a Polish case study
Source: BMC Vet Res. 2026 Mar 9;22:231. doi: 10.1186/s12917-026-05341-x (PMC13085543; doi:10.1186/s12917-026-05341-x)
Supplement: Supplementary file 1 — Additional file 1: Description of the laboratory method used to screen the broiler flocks. [file 12917_2026_5341_MOESM1_ESM.docx]

Laboratory screening methodology

The farm personnel on site organised each flock screening as part of their routine health management processes, independent of the laboratory. The process was based on the collection of two types of samples. First, the selection of five birds used in the screening (all birds were humanely euthanized before transport). Second, 23 birds were selected for blood samples drawn at the farm. All samples were collected from birds at the end of the production cycle, approximately 6 weeks of age, and sent to the veterinary laboratory by the farm’s means (no information on means and delay between sample collection and reception is known).

The registration of samples, their barcoding and the preparation of orders for microbiological, serological and Real-Time RT-PCR analysis took place at the ‘sample reception point’ of the laboratory SLW BIOLAB s. c. After barcoding, the samples were transferred to the appropriate Laboratory unit: the blood went to the ‘Serology’ unit, while the birds went to the dissection room of the ‘Microbiology’ unit. In the ‘Microbiology’ unit, in addition to securing material for microbiological examination, material from birds was also secured for Real-Time RT-PCR tests.

## Serological tests.

Blood samples in 1.5 ml Eppendorf-type centrifuge tubes were centrifuged in a Jouan centrifuge B4i multifunction (Thermo) at 2000 G for 5 minutes. The separated serum was transferred into clean tubes. Serum samples were stored in the freezer (-20 degrees) until the day of testing. For serological testing, tests from two manufacturers were used: IDEXX or BioCheck, following the manufacturers' recommendations for these tests. The degree of absorption of the test samples and controls was measured and recorded using a BioTek ELISA ELx800 reader at a wavelength of 650 nm for IDEXX and 405 nm for BioCheck. Determinations of specific antibody levels to avian metapneumovirus (aMPV), infectious bronchitis virus (IBV) and infectious bursal disease virus (IBDV) were performed using these tests.

## Anatomopathological, parasitological and microbiological examinations.

### Anatomopathological studies.

Birds were transferred to the post-mortem room of the Microbiology unit for description of anatomopathological changes and collection of organs for further examination. The examination order was also reviewed here, and the condition of the specimens was assessed.

The birds were laid on their backs on the dissecting table, and an external inspection was made, during which the following were assessed: plumage and skin surface, comb and wattle, ear lobe, eyes, beak and nasal openings, breast and limbs. If swellings of the ankle joint area were found, the joint capsules were incised, the contents of the ankle joint cavity were assessed and described, including their amount, colour and consistency. When pathological lesions were found in the ankle joints, cultures were performed on appropriate media.

The birds were then dissected. First, the skin behind the sternum was incised and dissected, allowing the pectoral muscles and leg muscles to become visible. The skin was then cut between the limb and the abdominal integuments on each side, after which the femoral head was assessed. If necrosis of the femoral head was found, cultures were taken from the femoral marrow into appropriate microbiological media. The body cavity was then opened with a transverse abdominal cut just behind the sternum, the sternum was tilted upwards, and the visible internal organs were assessed: the heart in the pericardial sac, liver, air sacs, kidneys, ureters, spleen and bursa of Fabricius. If lesions were present on the air sacs, cultures were performed at autopsy on microbiological media. Organs were then collected for microbiological examination: trachea, lungs, heart, liver and spleen. The proventriculi and gizzard, duodenum, jejunum, cecum with iliac and rectum were dissected, and the contents and condition of the mucous membranes were assessed. During visual inspection of the gastrointestinal tract, intestinal sections were taken for parasitological examination, and the duodenum for microbiological examination. The collected organs were transferred to a sterile glass plate to the Microbiology unit for microbiological culturing. After the birds were dissected, swabs from the palatal fissure and trachea, as well as organ sections (caecal tonsils, bursa of Fabricii) were collected for Real-Time RT-PCR. These samples were submitted to the PCR unit.

### Parasitological examination of intestinal mucosal scrapings.

From each bird, scrapings were taken for examination from four sections of the intestines: duodenum, small intestine (Meckel's diverticulum area), cecum and rectum.

After dissecting the relevant section of intestine, the contents were removed from the mucosal surface with the blunt part of the scissors in such a way as not to disturb the epithelium. The mucosal scraping was then removed with the edge of a coverslip, and the coverslip was transferred to a previously described slide. Immediately after taking the slide, the scrapings were viewed under a microscope (Zeiss Primostar 3) using three achromatic objectives at 10x, 20x and 40x magnification. The visible Eimeria spp. oocysts were counted from the entire surface of the slide, and the number of oocysts was determined, according to the criteria shown in Table 1.

**Table 1: Criteria for the interpretation of parasitological findings.**

| **Number of oocysts of Eimeria spp.**  **over the entire surface of the preparation** | **Interpretation of the result** | |
| --- | --- | --- |
| 0 | none | 0 |
| 1 - 9 | single | 1 |
| 10 - 50 | Moderately numerous | 2 |
| > 50 | numerous | 3 |

### Microbiological tests

In the Microbiology unit, cultures of internal organs were performed on microbiological media. Before starting the culture procedure, the surface of the organs placed on the plates was heated using a gas burner to minimise the risk of contamination of the cultures with microorganisms from the environment. Using sterile instruments, a section of the organ to be inoculated was cut to reveal its cross-section, the cross-sectional area was imprinted (imprint area of approximately 1 cm2) on a plate with the appropriate agar medium, and the reduction culture was performed according to the scheme presented in Table 2. In the case of Wrzosk liquid medium, the organ section was placed in a tube. The microbial media were then incubated for 24-48 h at 37 +/- 1°C (McConkey agar, Wrzosk liquid medium) or at 37 +/- 1°C under microaerophilic conditions - 5% CO_2_-enriched atmosphere (blood agar, Edwards agar).

**Table 2: Microbiological culture scheme:**

| **The organ** | **Microbiological media used for testing** |
| --- | --- |
| suborbital sinuses | - blood agar  - McConkey agar |
| trachea |  |
| lungs | - blood agar  - McConkey agar  - Edwards agar |
| heart |  |
| spleen | - blood agar  - McConkey agar |
| liver | - blood agar  - McConkey agar  - the liquid base of the Heather |
| duodenum |  |
| ankle joints | - blood agar  - Edwards agar |
| bone marrow |  |
| air sacs | - blood agar |

**Table 3: List of microbiological media used:**

| **Name of the medium** | **Part no., Manufacturer** |
| --- | --- |
| Agar with blood | - TSA PS 22-500, Graso Zenon Sobiecki  - sheep blood SL0160-500, BioMaxima S.A. |
| McConkey | PS10-500, BioMaxima S.A. |
| Edwards | CM0027B, Argenta Sp. z o. o. Sp. K. |
| Wrzosek | - Schaedler broth PS 19-500, BioMaxima S.A.  - dried liver D-086, BTL Sp. z o. o. **Enzymes and Peptones Plant**  - Paraffin D-037, BTL Sp. z o. o. **Enzymes and Peptones Plant** |

Microbiological reading of the inoculated media was performed twice: after an overnight incubation and after 48 hours of incubation. Each medium and seeded organ was analyzed for the types of pathogenic bacteria and their abundance defined in the four following categories:

- Single (1): up to 10 colony-forming units per plate,
- Medium abundant (2): 11-100 colony-forming units per plate,
- Numerous (3): more than 100 colony-forming units per plate,
- Not defined (ND): micro-organism count not determined, culture method consisting of pregrowth of material to isolate anaerobic bacteria (applies to bacteria that were cultured from Wrzoska medium).

For the detection of anaerobic bacteria, after 24 h incubation, the tubes of Wrzosk medium that showed turbidity or visible gas bubbles were sieved onto blood agar for the culture of anaerobic bacteria (including Clostridium). These media were incubated at 37 +/- 1°C for 24 h in an anaerobic atmosphere using anaerogen jars (OXOID, AnaeroGen™ 2.5 L). The number of microorganisms cultured by this method was not determined; they were labelled ‘ND’ in the study report.

According to the criteria, the number of colonies that were morphologically different from the others was determined. Isolations were then performed on non-selective blood agar medium to multiply the colonies for further studies, and the cultures were incubated for 24 h at 37 +/- 1°C under aerobic, microaerophilic, or anaerobic conditions, depending on the microorganism. After incubation, the identification of isolated bacteria was performed by MALDI-TOF mass spectrometry (MALDI-TOF MS, Biotyper, Bruker). From the identified bacterial colonies, a drug susceptibility determination of the microorganism was performed using the disk diffusion method. The number of colonies used for the test was such that the final suspension of bacteria in sterile deionised water (SM) reached a turbidity of 0.5 McFarland degree. The standardised inoculum was plated onto Mueller-Hinton agar plates (Mueller-Hinton II agar, REF 116 Graso Zenon Sobiecki) or Mueller-Hinton blood agar (Mueller-Hinton II agar, REF 116 Graso Zenon Sobiecki, sheep blood SL0160-500, BioMaxima S.A.) depending on the bacterial species.

Diagnostic discs were then applied to the surface of the medium using special dispensers, and the plates were incubated for 16-18 h or 20-24 h, depending on the type of bacteria, at 35 +/- 2°C.

After the prescribed incubation time, the antibiograms were read. Measurements were taken under transmitted light by measuring the zone of inhibition of microbial growth around the diagnostic discs with a calliper. The zone diameter value in mm, as measured by the callipers, was transferred to a computer program, which automatically recalculated the numerical values for the zones of inhibition. In this way, an interpretation of drug susceptibility was obtained: 0 - resistant, 1 - moderately sensitive or 2 sensitive. The laboratory uses a proprietary computer program.

**Table 4: List of antibiotic discs used**

| **Name** | **Part no., Manufacturer** |
| --- | --- |
| Amoxycillin | CT0061B, OXOID |
| Amoxycillin/Clavulanic acid | CT0223B, OXOID |
| Colistin Sulphate | CT0017B, OXOID |
| Doxycycline | CT0018B, OXOID |
| Enrofloxacin | CT0639B, OXOID |
| Florfenicol | CT1754B, OXOID |
| Flumequine | CT0666B, OXOID |
| Lincomycin | CT0028B, OXOID |
| Lincomycin /Spectinomycin | CT1758B, OXOID |
| Neomycin | CT0033B, OXOID |
| Oxytetracycline | CT0041B, OXOID |
| Compound Sulphonamides | CT0059B, OXOID |
| Sulphamethoxazole/Trimethoprim | CT0052B, OXOID |
| Thiamulin | 9094, Liofilchem |
| Tylosin | 9082, Liofilchem |
| Phenoxymethylpenicillin 10 | E191712, BioMaxima S.A. |

## Detection of IBV (variant Var2, 4/91, QX, Mass, D274, IB80), aMPV (types A and B) and IBDV (VP2 of A1 or A3) virus RNA by Real-Time RT-PCR.

Cecal tonsils, upper respiratory tract and bursa of Fabricius were sampled to detect IBV, aMPV and IBDV, respectively. Prepared and pelleted cecal tonsil scrapings and bursae of Fabricius were suspended in physiological fluid and partially homogenised using glass beads. Upper respiratory tract swabs were bulked and washed in physiological fluid. Total viral genetic material from the prepared samples was isolated using a commercial MagMAX™ CORE Nucleic Acid Purification Kit (Thermo Fisher Scientific) and a KingFisher™ Duo Prime Purification System machine (Thermo Fisher Scientific).

The resulting product was used to detect the presence of RNA of IBV, aMPV and IBDV viruses by Real-Time RT-PCR. The analysis was performed using commercial Kylt® kits designed for the separate, specific detection of genetic material of IBV viruses (variant Var2, 4/91, QX, Mass, D274, IIB80, aMPV (types A and B) and IBDV (A1 or A3) using specific primers and fluorescently labelled probes. The list of kits used and catalogue numbers is shown in Table 5. Real-Time RT-PCR reactions were performed according to the manufacturer's protocol using a Bio-Rad 7500 CFX96 Real-Time System thermocycler (Bio-Rad) under the conditions outlined in Table 6. Fluorescence results were read using dedicated CFX Manager Dx software (BioRad).

**Table 5: Kylt® RT-PCR kits used in the experiment:**

| **Set name** | **Catalogue no.** |
| --- | --- |
| Kylt^®^ IBV-Variant 02 | 31187 |
| Kylt^®^ IBV-Variant 4/91 | 31082 |
| Kylt^®^ IBV-Variant QX | 31094 |
| Kylt® IBV-Variant Massachusetts | 31084 |
| Kylt^®^ IBV-Variant D274 | 31086 |
| Kylt^®^ IBV-Variant IB80 | 31736/7 |
| Kylt® aMPV A&B | 31072 |
| Kylt® IBDV Typing | 31443 |

**Table 6: Real-Time -RT-PCR reaction conditions used to detect genetic material of individual viruses:**

|  | **Temperature profile for aMPV and IBDV** | | | **Temperature profile for IBV**  **variants** | | |
| --- | --- | --- | --- | --- | --- | --- |
| **Steps** | Temperature | Duration | Number of cycles | Temperature | Duration | Number of cycles |
| **Reverse transcription** | 50°C | 10 min |  | 50°C | 10 min |  |
| **Polymerase activation** | 95°C | 1 min |  | 95°C | 1 min |  |
| **Denaturation** | 95°C | 10 sec | 42 cycles | 95°C | 10 sec | 42 cycles |
| **Attachment of genetic material** | 60°C | 1 min |  | 55°C | 1 min |  |
